# Genome-wide DNA methylation profile identified a unique set of differentially methylated immune genes in oral squamous cell carcinoma patients in India

**DOI:** 10.1186/s13148-017-0314-x

**Published:** 2017-02-03

**Authors:** Baidehi Basu, Joyeeta Chakraborty, Aditi Chandra, Atul Katarkar, Jadav Ritesh Kumar Baldevbhai, Debjit Dhar Chowdhury, Jay Gopal Ray, Keya Chaudhuri, Raghunath Chatterjee

**Affiliations:** 1 Human Genetics Unit, Indian Statistical Institute, 203 B. T. Road, Kolkata, 700108 India; 2 Molecular Genetics Division, CSIR-Indian Institute of Chemical biology, 4 Raja S C Mullick Road, Kolkata, 700 032 India; 3 Dr. R Ahmed Dental College & Hospital, 114, A J C Bose Road, Kolkata, India

**Keywords:** Oral squamous cell carcinoma, CTLA4, CpG island, Differentially methylated regions, Methylation-specific PCR, Bisulfite cloning and sequencing, Gene expression

## Abstract

**Background:**

Oral squamous cell carcinoma (OSCC) is one of the common malignancies in Southeast Asia. Epigenetic changes, mainly the altered DNA methylation, have been implicated in many cancers. Considering the varied environmental and genotoxic exposures among the Indian population, we conducted a genome-wide DNA methylation study on paired tumor and adjacent normal tissues of ten well-differentiated OSCC patients and validated in an additional 53 well-differentiated OSCC and adjacent normal samples.

**Results:**

Genome-wide DNA methylation analysis identified several novel differentially methylated regions associated with OSCC. Hypermethylation is primarily enriched in the CpG-rich regions, while hypomethylation is mainly in the open sea. Distinct epigenetic drifts for hypo- and hypermethylation across CpG islands suggested independent mechanisms of hypo- and hypermethylation in OSCC development. Aberrant DNA methylation in the promoter regions are concomitant with gene expression. Hypomethylation of immune genes reflect the lymphocyte infiltration into the tumor microenvironment. Comparison of methylome data with 312 TCGA HNSCC samples identified a unique set of hypomethylated promoters among the OSCC patients in India. Pathway analysis of unique hypomethylated promoters indicated that the OSCC patients in India induce an anti-tumor T cell response, with mobilization of T lymphocytes in the neoplastic environment. Survival analysis of these epigenetically regulated immune genes suggested their prominent role in OSCC progression.

**Conclusions:**

Our study identified a unique set of hypomethylated regions, enriched in the promoters of immune response genes, and indicated the presence of a strong immune component in the tumor microenvironment. These methylation changes may serve as potential molecular markers to define risk and to monitor the prognosis of OSCC patients in India.

**Electronic supplementary material:**

The online version of this article (doi:10.1186/s13148-017-0314-x) contains supplementary material, which is available to authorized users.

## Background

Oral cancer consists of any malignant neoplasm in the lip, floor of the mouth, buccal mucosa, gingiva, retromolar trigone, palate, or in the tongue and is the most common form of cancer in the head and neck squamous cell cancer (HNSCC) category. It is the 11th most common cancer worldwide with an estimated 300,400 new cases and 145,400 deaths occurred in 2012 worldwide [1]. Globally, India has the highest incidence of oral cancer per year and is the leading cancer among Indian men and fifth most common among women [2, 3]. Most oral malignancies occur as squamous cell carcinomas (SCCs), and many OSCCs develop from premalignant conditions of the oral cavity [4]. A wide array of conditions have been implicated in the development of oral cancer, including leukoplakia, erythroplakia, palatal lesion of reverse cigar smoking, oral lichen planus, oral submucous fibrosis, discoid lupus erythematosus, and hereditary disorders such as dyskeratosis congenital and epidermolysis bullosa [5]. Despite the general accessibility of the oral cavity during physical examination, many malignancies are not diagnosed until late stages of the disease. The major risk factors for oral cavity cancer are smoking, smokeless tobacco, alcohol, and HPV infection [1]. The higher incidence of oral cancer in India, Taiwan, and other neighboring countries is attributed to popularity of different oral habits like chewing smokeless tobacco, betel quid, and areca nut [1].

Oral cancer is a multifactorial disease involving genetic and epigenetic abnormalities. The addition of a methyl group at the cytosine residue of CpG dinucleotide have a profound effect on initiation and progression of cancer [6–9]. Hypermethylation of CpG islands (CGIs) in the promoter region results in transcriptional silencing of tumor suppressor genes, whereas hypomethylation leads to oncogene activation in many cancers [7–10]. As DNA methylation alteration often occurs early in cancer development, candidate methylation markers may be valuable for early and specific cancer detection [11–13]. Promoter hypermethylation-mediated silencing of different classes of genes involved in cell-cycle regulation, signaling pathways, angiogenesis, proliferation, differentiation, DNA repair, and apoptosis are reported in OSCC [13–22].

Studies have indicated involvement of genetic, epigenetic, and oral habits in the development of oral cancer. The contribution of the varied risk factors in the Indian subcontinent may lead to a distinct disease pathogenesis. The mechanisms by which different habits influence phenotype and disease risk may involve the altered epigenetic regulation of genes. Recently, candidate gene-specific studies identified epigenetic modifications in the promoter regions of OSCC patients in India. *P16*, *DAPK*, and *MGMT* gene promoter hypermethylation was reported in oral cancer tissues compared to corresponding adjacent normal mucosa [23]. *EDNRB*, *KIF1A*, *DCC*, *P16*, *P15*, *hMLH1*, *MGMT*, and E-cadherin gene promoter hypermethylation is also observed in Indian OSCC patients [24, 25]. Studies with esophageal cancer patients from North Eastern Indians have shown that tobacco consumption possibly interacts with carcinogen-metabolizing genes and modulate the promoter hypermethylations of tumor suppressor genes [26]. However, most of these studies focused on cancer-associated hypermethylation at specific genes. Surprisingly, OSCC-associated DNA hypomethylation received little attention. Furthermore, global hypomethylation observed in OSCC have not been characterized profoundly among Indian patients.

High throughput genome-wide methylation study provides a comprehensive platform to understand the overview of DNA methylation [27]. Genome-wide DNA methylation profiles in the OSCC patients of this region thus may help us to understand the disease pathogenesis. However, to date, there is no systematic genome-wide study to investigate whether changes in DNA methylation can influence the OSCC development among the patients in India. In the present study, we have determined the global methylation status of well-differentiated OSCC and adjacent normal tissues using Illumina Infinium 450K BeadChip Array. Differentially methylated regions were validated in another cohort of OSCC patients in India. Gene expression profiles of some of these aberrantly methylated genes showed inverse correlation with the promoter methylation. A comparative analysis of the methylome data of these OSCC patients with the available TCGA HNSC patients identified some novel genes, which showed exclusive differential methylation in OSCC patients of Indian population. Survivability analysis suggested prognostic significance of some of these genes in OSCC.

## Results

### Genome-wide DNA methylation distribution in the well-differentiated OSCC

**Fig. 1:**
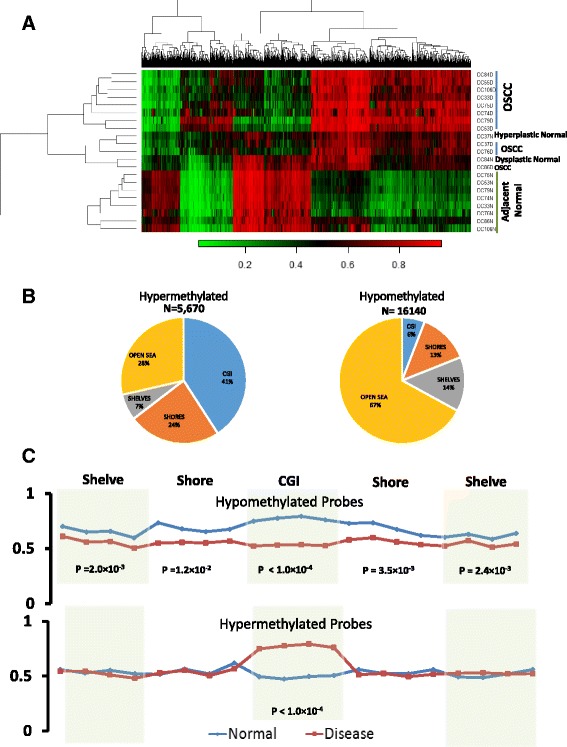
Distribution of differentially methylated probes (DMPs). **a** Heatmap of top 2000 DMPs. Hierarchical clustering showing separate grouping of DMPs for OSCC and adjacent normal tissue. **b** Genomic localisation of hypermethylated and hypomethylated probes at the CpG islands (CGIs), shore, shelves, and open sea. **c** Average methylation across the CGIs, shores, and shelves for hypo- and hypermethylated CGIs

We have determined the genome-wide DNA methylation profiles of OSCC patients using the Illumina Infinium 450K array. In total, 64 OSCC patients were recruited for the study (Table 1, Additional file 1: Table S1). All tissue samples were histopathologically verified and diagnosed to be well-differentiated OSCC. Ten pairs of cancer and adjacent normal tissues and one unpaired cancer tissue from the buccal mucosa of OSCC patients were used for the global methylome analysis. To avoid the influence of DNA methylation on X chromosome inactivation, we considered only the autosomal probes for further analysis. Probes showing |Δβ| ≥0.20 between the cancer and adjacent normal tissues with adjusted *P* value ≤0.05 were defined as differentially methylated probes (DMPs). Following the criteria, we identified 21,810 DMPs including 16,140 hypomethylated and 5670 hypermethylated probes. Hierarchical clustering of top 2000 DMPs showed separate clustering of OSCC and adjacent normal tissues (Fig. 1a), except for two normal samples which clustered with disease group. This might be due to hyperplastic to mild dysplastic cells, as noticed in the histopathology of these samples (Additional file 2: Figure S1A–G). However, no distinct clustering was observed between HPV-positive and HPV-negative samples. Separate comparison between HPV-positive and HPV-negative samples could not identify any probe that was significantly differentially methylated according to our criterion |Δβ| ≥0.20 and adjusted *P* value ≤0.05.

**Table 1:** Demographic and clinical characteristics of the discovery and validation set

Characteristics	Patient information	
	Discovery set	Validation set
Total patients	11	53
Normal/disease	10/11	53/53
Age range (in years)	40–90	27–78
Mean age (SD) (in years)	51.37 (14.33)	53.50 (12.45)
No. of males/females	7/4	39/14
HPV: positive/negative	6/5	29/24
Oral habits		
Smoking tobacco	1	2
Chewing tobacco	2	22
Other habits	1	1
Multiple habits	4	10
No habits	2	18

Next, we sought to determine the enrichment of 21,810 DMPs at CpG islands (CGIs) and non-CGI regions of the genome (Additional file 2: Figure S2A, B). Enrichment of hyper- and hypomethylated probes were significantly different between the CGIs and non-CGI regions (Additional file 2: Figure S2A, B). Approximately, 85% of the DMPs were in the non-CGI, and majority (82%) of these probes are hypomethylated. On the contrary, majority of the CGI-overlapped DMPs (70%) are hypermethylated (Additional file 2: Figure S2A, B). Further classification of these DMPs based on their enrichment at CGIs, shores, shelves, and open sea clearly showed that majority of the hypermethylated probes (72%, i.e., 4082 DMPs) were enriched in the CGIs, shores, and shelves, whereas hypomethylated probes (67%) were mostly enriched in the open sea (Fig. 1b). To determine the genomic localization of these probes, we mapped these DMPs into the promoters, exons, introns, repeat regions, and other regions (Additional file 2: Figure S2C, D). Complying with the previous observations, we found that the CGI-overlapped DMPs were mostly hypermethylated and the non-CGI DMPs were mostly hypomethylated irrespective of their genomic localizations. The pattern of differential methylation across the CGIs, shores, and shelves showed contrasting characteristics between hypomethylated and hypermethylated regions. The hypermethylation of the CpGs were specifically localized to the CGIs (*P* value ≤0.0001), while shores and shelves did not show any significant differential methylation (*P* value ≥0.05). On the contrary, significant hypomethylation (*P* value ≤0.05) was observed across CGIs, shores, and shelves between tumors and adjacent normal tissues, suggesting different mechanisms of hypo- and hypermethylation in OSCC development (Fig. 1c). The hierarchical clustering of average β values for the promoter and the CGIs also indicated the distinct epigenetic regulations between the well-differentiated OSCC and adjacent normal tissues (Additional file 2: Figure S3A, B).

### Validation of differentially methylated promoters

**Fig. 2:**
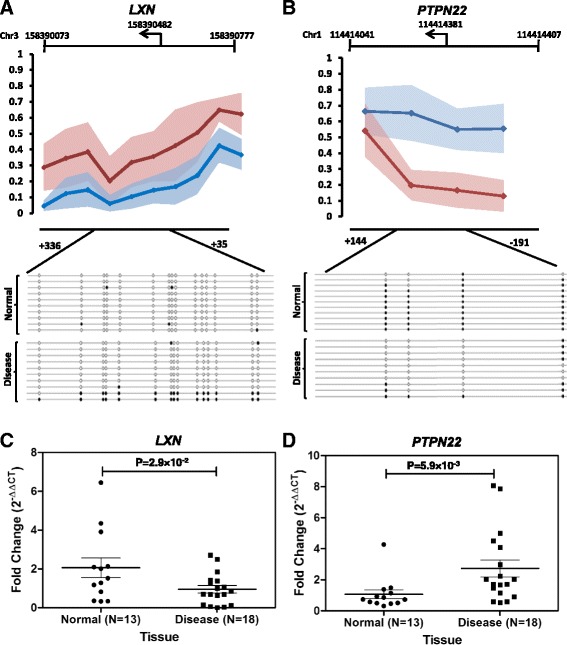
Validation of HumanMethylation450K bead chip data. The *upper panel* shows the avergae β values, and the *lower panel* shows the clonal validation by sequencing the bisulfite-converted products of **a** LXN and **b** PTPN22 promoters. The *filled circle* in the *lower panel* represents the methylated, and the *open circle* represents the unmethylated CpGs. Gene expression of **c** LXN and **d** PTPN22 showed significant differential expression between OSCC and adjacent normal tissues, correlating with methylation status

In order to validate the 450K array results, we determined the methylation status of five randomly selected differentially methylated promoters by sequencing the cloned bisulfite-converted DNA samples of OSCC and adjacent normal tissues (Fig. 2a, b, Additional file 2: Figure S4). Clonal validation showed hypermethylation for the *HLA-DPB1* (12 to 81%), *LDLRAD4* (0 to 38%), *LHX1* (34 to 98%), and *LXN* (2 to 29%) promoters and hypomethylation for *PTPN22* (86 to 30%) in tumors compared to adjacent normal tissues. These results were consistent with that of the 450K array data. Furthermore, a panel of 14 differentially methylated promoters were selected for validation in additional 53 pairs of tumor and adjacent normal tissues using methylation-specific quantitative real-time PCR (qMSP) (Table 2). Most of the differentially methylated promoters showed 60 to 90% concordance with the results obtained from the genome-wide methylation data. Some of the discordant results can also be attributed to the hyperplastic or dysplastic tissue morphology on some of the adjacent normal samples.

**Table 2:** Validation of differentially methylated regions

Methylation status in 450K array	Overlap with CGI	Gene	No. of samples	No. of concordant samples	% of concordant samples
Hypermethylated	CGI	*ZNF577*	53	42	79.25
		*HLA-DPB1*	51	38	74.51
		*LHX1*	53	41	77.36
		*MYH14*	13	11	84.62
	Non-CGI	*ZSCAN31*	47	35	74.47
		*LDLRAD4*	53	37	69.81
		*LXN*	53	37	69.81
Hypomethylated	CGI	*PIWIL1*	14	13	92.86
		*MFAP2*	11	9	81.82
	Non-CGI	*PTPN22*	53	33	62.26
		*SPNS3*	14	10	71.43
		*AIM2*	12	10	83.33
		*SLAMF1*	12	8	66.67
		*SPATA13*	10	7	70.00

### Transcriptional effects of aberrant DNA methylation

To investigate the transcriptional regulation of differentially methylated promoters in OSCC compared to adjacent normal tissues, we studied gene expression profile of 14 differentially methylated genes identified from the discovery cohort (Fig. 2c, d, Additional file 2: Figure S5, 6). These included seven genes with hypermethylated promoters, namely, *LXN*, *ZNF154*, *ZNF577*, *ZSCAN31*, *CTDSP1*, *LDLRAD4*, and *HLA-DPB1*, and seven with hypomethylated promoters, including *PTPN22*, *RUNX1*, *IL6*, *CD28*, *TLR1*, *CD80*, *CD22*, and *TNFa*. Genes with hypermethylated promoters such as *LXN* (*P* value = 2.94 × 10^−2^), *ZNF154* (*P* value = 3.10 × 10^−2^), *CTDSP1* (*P* value = 2.40 × 10^−2^), and *ZNF577* (*P* value = 1.80 × 10^−3^) showed significant downregulation in OSCC tissues compared to the adjacent normal tissues (Fig. 2c, d, Additional file 2: Figure S5). *ZSCAN31* and *LDLRAD4* showed downregulation but did not reach the level of significance at 0.05. Only, *HLA-DPB1* showed significant upregulation (*P* value = 3.80 × 10^−2^) in spite of its promoter getting hypermethylated. A closer look into the methylation data showed hypomethylation at the promoter region of another isoform of the *HLA-DPB1* gene, which may be the reason for this discordant expression profile. Similarly, significant upregulations were observed for the hypomethylated genes *PTPN22* (*P* value = 5.9 × 10^−3^), *RUNX1* (*P* value = 4.5 × 10^−3^), *IL6* (*P* value = 1.56 × 10^−2^), *CD28* (*P* value = 7.5 × 10^−3^), *TLR1* (*P* value = 2.63 × 10^−2^), *CD80* (*P* value = 3.34 × 10^−5^), and *TNFa* (*P* value = 9.8 × 10^−3^) (Fig. 2d, Additional file 2: Figure S6). The gene expression analysis was consistent with methylation status and further showed the influence of differential promoter methylation in regulating the transcriptional activity of the genes. This also strengthened the validity of the differentially methylated genes identified in our discovery cohort.

### Comparison of methylation changes with TCGA data

**Fig. 3:**
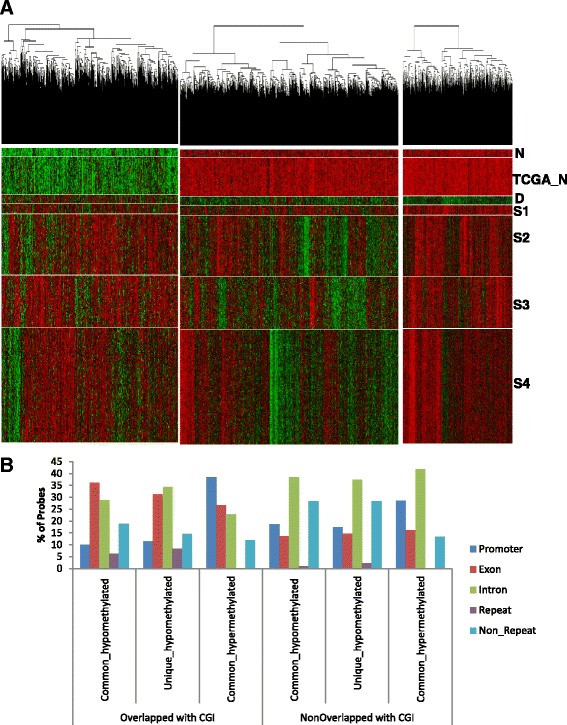
Comparison of HumanMethylation450K bead chip with TCGA HNSC samples. **a** Hierarchical clustering of common hypermethylated, common hypomethylated, and unique hypomethylated probes in OSCC (D) and adjacent normal samples in India (N), and TCGA normal (TCGA_N) and HNSC samples for stages 1 (S1), 2 (S2), 3 (S3), and 4A (S4). **b** Genomic localization at the promoters, exons, introns, repeat regions, and other regions of common hypomethylated, hypermethylated, and unique hypomethylated probes that are overlapped with CGIs and non-CGIs

We compared the genome-wide DNA methylation data of the OSCC patients in India with that of the available TCGA HNSC samples (https://tcga-data.nci.nih.gov/docs/publications/tcga/?). We only considered 312 samples at stages 1, 2, 3, and 4A among the 526 publicly available TCGA HNSC samples (Additional file 3: Table S2). As we do not have any patients with malignancies at the larynx, alveolar ridge, oropharynx, and hypopharynx and tonsil carcinoma, remaining 215 TCGA samples with malignancies at these regions were not included in our comparative analysis. Hazard ratio was estimated in Cox model based on the death due to oral cancer by age, tumor stage, and node involvement. Significant risk was observed only for the age at the initial pathological diagnosis of OSCC patients, while tumor stages and node involvement status showed marginal increased risk but did not reach the level of significance at 0.05 (Additional file 3: Table S3). Approximately, 92.7% (14,975) of the hypomethylated and 100% of the hypermethylated probes had similar methylation pattern, at least with one stage of the TCGA samples. Interestingly, a subset of differentially methylated probes (1165) were uniquely hypomethylated in tumor tissues of OSCC patients in India. The methylation profile of these probes were significantly different (adjusted *P* value ≤0.05) from all stages of the TCGA data (Fig. 3a, Additional file 2: Figure S7–9). We determined the genomic distribution of the common and unique DMPs. We observed that the hypermethylated probes at the CGIs were mainly enriched at the promoters, while hypomethylated probes at the CGIs were primarily enriched at the exons and introns (Fig. 3b, Additional file 2: Figure S2B, C). On the contrary, both hypo- and hypermethyated probes at the non-CGIs are mostly enriched in the introns. Among the 1165 unique DMPs, 162 probes overlapped with promoter region of 134 genes. Among these, 40 promoters were overlapped with CGI, while 94 were non-CGI-associated promoters. (Additional file 4: Table S4).

### Gene ontology enrichment and IPA analysis of differentially methylated promoters

**Fig. 4:**
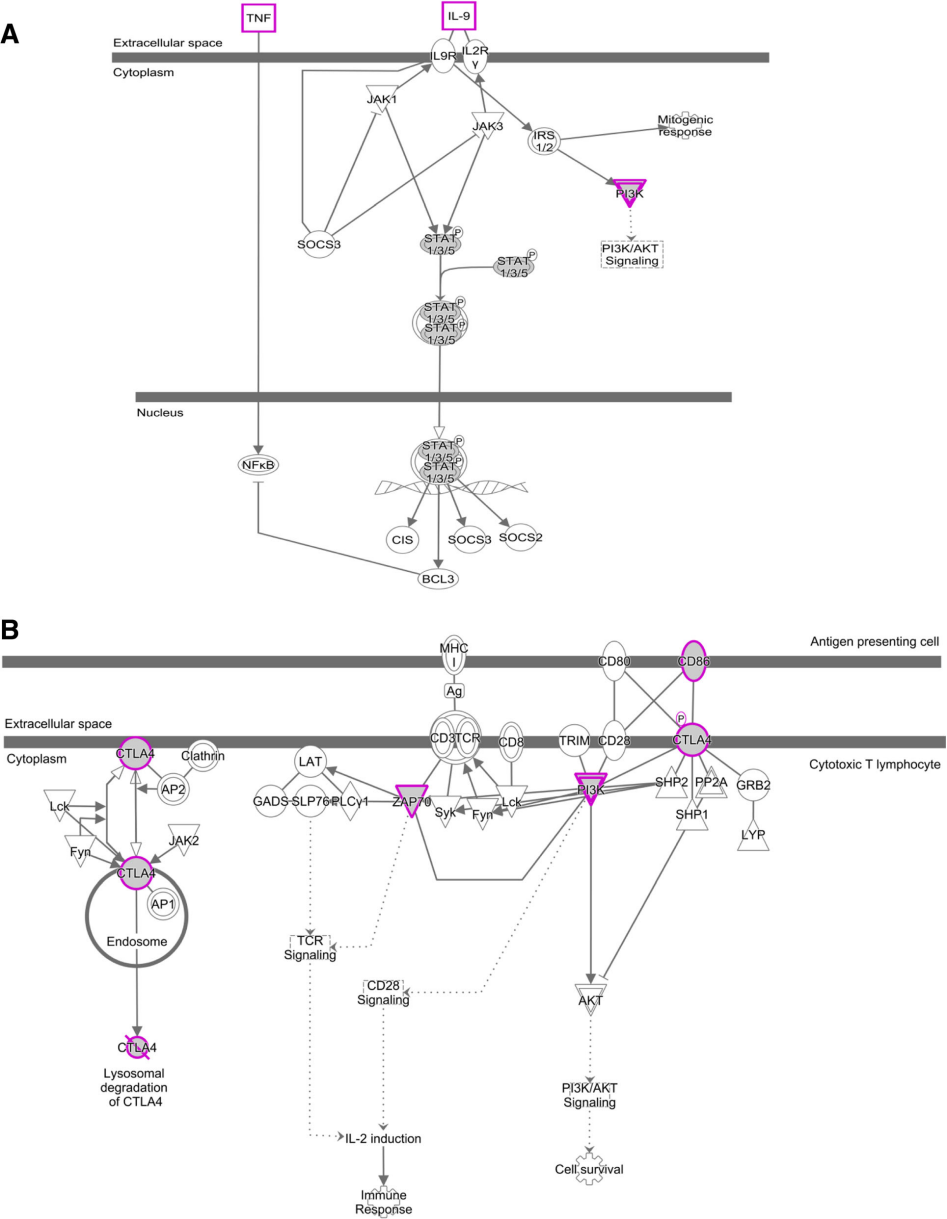
Ingenuity pathway analysis of uniquely hypomethylated promoters. The most enriched pathways are **a** IL9 signaling pathway and **b** CTLA4 signaling in cytotoxic T lymphocytes. Common hypomethylated genes are overlapped with these pathways. *Gray-shaded* genes are common hypomethylated, and *pink-enclosed* genes are with unique hypomethylated promoters

**Fig. 5:**
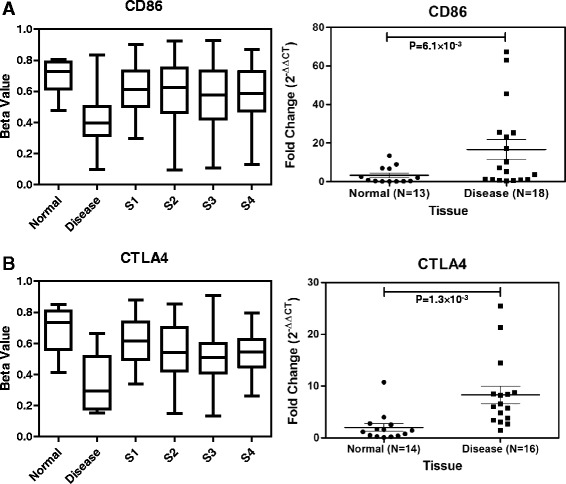
Comparison of uniquely hypomethylated probe between human methylation 450K bead chip and TCGA samples. Methylation status of unique probes on **a** *CD86* and **b** *CTLA4* promoter in OSCC (disease) and adjacent normal samples (normal) in India and different stages of TCGA samples (S1, stage 1; S2, stage 2; S3, stage 3; and S4, stage 4A). Methylation profile of these genes in disease tissue of Indian patients was significantly different from their normal counterpart, as well as from disease stages S1, S2, S3, and S4A of TCGA samples. Gene expression (fold change) of *CD86* and *CTLA4* showed significantly higher expression in OSCC compared in the adjacent normal tissues among Indian samples. *P* value **≤**0.05 was considered significant

To determine the functional implication of the common and unique DMPs, we performed gene ontology analysis. In total, 21 biological processes have been enriched at least by 1.5-fold with enrichment *P* value ≤0.01 for the common hypermethylated promoters (Additional file 2: Figure S10). Majority of the enriched biological processes are related to regulation of transcription, embryonic morphogenesis, cell signaling, transmission of nerve impulse, and multicellular organismal response to stress. Among the common hypomethylated promoters, the major biological processes are mainly related to sensory perception like sensory perception of chemical stimulus and cognition, inflammatory response, G-protein-coupled receptors, and metal ion transport. Twelve biological processes are significantly enriched for the unique hypomethylated promoters. These are immune response, positive regulation of cytokine biosynthesis, regulation of cell proliferation, T cell activation, and etc. Enrichment of these immune response genes indicates infiltration of immune cells into the tumor microenvironment. Our discovery cohort consists of both HPV-positive and HPV-negative OSCC samples. We did not find any differential methylation for these immune genes between OSCC and adjacent normal tissues, suggesting that the unique hypomethylation in these immune genes are not consequence of HPV infection. To determine the pathways that may be effected by the unique hypomethylated promoters, we performed ingenuity pathway analysis. The top significantly enriched canonical pathways are the IL9 signaling and CTLA4 signaling in cytotoxic T lymphocytes (Fig. 4). *CTLA4* activation upon promoter hypomethylation indicates the negative regulation of effector T cell activation. Overlapping the common hypomethylated promoter with the CTLA4 signaling pathway suggested preferential binding of CD86 with CTLA4 than CD28 to negatively regulate the cytotoxic T cell activation in the neoplastic environment (Fig. 4b). CD28 promoter is hypomethylated in both TCGA and OSCC patients in India and found to be upregulated in the OSCC tissues compared in the adjacent normal tissues (Additional file 2: Figure S6D, S8B). We determined the gene expression profile of both *CD86* and *CTLA4* in the OSCC and adjacent normal tissues (Fig. 5). Both *CD86* (*P* value = 6.10 × 10^−3^) and *CTLA4* (*P* value = 1.30 × 10^−3^) showed significant overexpression in the OSCC tissues compared in the adjacent normal tissues, which further validated the epigenetic regulation of *CD86* and *CTLA4*, specifically among the OSCC patients in India.

### Clinical significance of epigenetically regulated immune genes

**Fig. 6:**
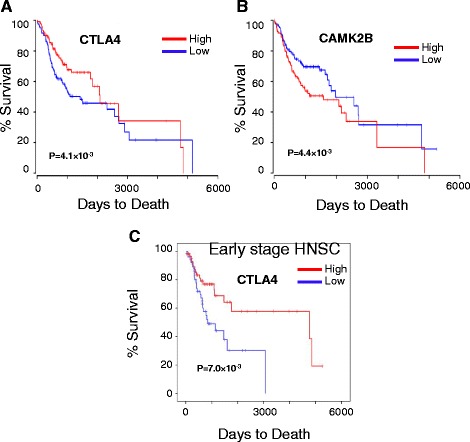
Clinical significance of genes associated with uniquely hypomethylated probes. Kaplan-Meier plots of **a** *CTLA4* showed higher gene expression (high, *N* = 124) was significantly associated with better disease prognosis than low expression (low, *N* = 124), while **b** lower expression of *CAMK2B* was associated with better prognosis. **c** Early-stage patients (stages 1 and 2) showed significantly better prognosis with higher expression of *CTLA4*. Mantel-Cox log-rank *P* values are presented with each survival analysis

We next focused on the clinical significance of 134 unique differentially methylated genes observed in our study. The survival analysis based on the top 30 unique DMPs failed to show any significant association with clinical outcome of the TCGA HNSC patients. However, expression profile of 17 unique differentially methylated genes showed significant prognostic relevance in HNSC. Higher expression of *CTLA4* (*P* value = 4.06 × 10^−2^), *CCR10* (*P* value = 8.94 × 10^−3^), *SLAMF1* (*P* value = 4.85 × 10^−3^), *SLC26A1* (*P* value = 1.08 × 10^−2^), *TNFRSF4* (*P* value = 3.90 × 10^−4^), *APOBR* (*P* value = 2.58 × 10^−2^), *CACNA2D3* (*P* value = 1.42 × 10^−3^), *CLNK* (*P* value = 4.77 × 10^−4^), *BFSP2* (*P* value = 8.79 × 10^−4^), *FCRL6* (*P* value = 6.14 × 10^−3^), *MFSD7* (*P* value = 3.52 × 10^−2^), *P2RY14* (*P* value = 1.67 × 10^−4^), *TLR9* (*P* value = 8.75 × 10^−3^), and *ZAP70* (*P* value = 1.28 × 10^−4^) was significantly correlated with better disease prognosis (Fig. 6, Additional file 2: Figure S11, 12). On the contrary, low expression of *CAMK2B* (*P* value = 4.42 × 10^−2^), *MTHFD1L* (*P* value = 2.27 × 10^−2^), *TKT* (*P* value = 3.96 × 10^−2^) was significantly associated with better survival (Fig. 6, Additional file 2: Figure S12). Ten genes, out of these 14 genes that showed significantly better prognosis with higher expression, are involved in immune response pathways (Fig. 6, Additional file 2: Figure S11E, F). The Kaplan-Meier test based on the gene expression profile of *CTLA4* showed significantly (*P* value = 0.007) better prognosis for the early-stage (stages 1 and 2) patients when they are overexpressed (Fig. 6), while late-stage (stages 3 and 4) patients failed to show any significant association (*P* value = 0.53) (Additional file 2: Figure S12G).

## Discussion

In this study, we determined the genome-wide methylation profile of well-differentiated OSCC and adjacent normal tissues. We identified several novel differentially methylated regions in oral cancer patients in India.

A total of 21,810 probes were identified to be differentially methylated in well-differentiated squamous cell carcinoma as compared to adjacent normal tissues. The analysis followed a stringent cut off of |Δβ| ≥0.2 and combined *P* value ≤0.05. DMPs were clustered in two distinct groups showing a clear difference in methylation pattern among disease and adjacent normal tissues of the same patients. Mis-clustering of the two normal samples were attributed to the hyperplastic to mild dysplastic features of these samples. Moreover, one of the patient was aged 90 years. The age of this patient was almost an outlier in the discovery set, and it may have modulated the epigenomic pattern of this patient. We found 25% of the DMPs were hypermethylated and the remaining 75% were hypomethylated. As observed in several cancers, we also found preferential enrichment of hypermethylated probes in the CpG-rich regions, while hypomethylated probes were primarily enriched at the open sea [28]. Interestingly, we have shown that the epigenetic drift at the CGIs, shores, and shelves were somewhat different for hyper- and hypomethylated. In case of hypermethylated promoters, no variation in the methylation pattern within the shore and shelves were observed. Only an abrupt and strong enrichment of hypermethylation in the CGI regions were observed. On the contrary, hypomethylated promoters showed hypomethylation getting spread towards the shore and shelves. This data suggests that two independent mechanisms of hypo- and hypermethylation are acting in OSCC development. It has recently been reported that the genome-wide hyper- and hypomethylation patterns are controlled by distinct sets of epigenetic enzymes, which is probably acting independent but simultaneously in two parts of the genome for hypo- and hypermethylation [29, 30].

Aberrant promoter methylation leading to transcriptional deregulation of a gene are often found in cancers. In the present study, we focused on the DMPs that overlapped with the promoter regions. We have validated a panel of 14 differentially methylated promoters, identified from the genome-wide methylation data, in another cohort of 53 well-differentiated OSCC samples. Most of the promoter regions have shown methylation pattern consistent with the array data. We identified several novel candidate genes that were deregulated due to aberrant promoter methylation in OSCC. Differential methylation in promoters as well as altered expression of *LXN*, *ZNF154*, *ZNF577*, *CTDSP1*, *RUNX1*, *CD28*, and *CD80* have not been reported previously in OSCC. *LXN* mainly functions as a potential tumor suppressor and reduces the stem cells transformation into cancer stem cell. It has previously been shown to be inhibited by promoter hypermethylation in malignant melanoma, gastric cancer, and prostate cancer [31–34]. *ZNF154* and *ZNF577* downregulation has been reported to be associated with several cancers [35, 36]. Inhibition of *CTDSP* family members promotes the G1/S-phase transition and have been found to be downregulated in hepatocellular carcinoma [37]. Recently, epigenetic regulation of *PTPN22* in the esophegal squamous cell carcinoma has been reported [38].We observed distinct hypomethylation of the *PTPN22* promoter in OSCC, which also had inverse correlation with expression pattern of the gene.

Oral cancer is one of the most common malignancies in India [2]. Environmental exposure to genotoxic agents such as betel quid, gutkha, chewing tobacco, smoking, and alcohol have been identified as risk factors towards the development of oral cancer [39, 40]. Although methanolic extract of *Piper betel* L. has been reported to have immunosuppressive effects [41], however, when consumed in combination with areca nut and slaked lime (betel quid), it can be harmful. Areca nut has been identified as an independent group I human carcinogen [42]. Areca nut chewed in different forms is predominant among the peoples of South and Southeast Asian countries [43]. Pan masala and gutkha, which have very high concentration of areca nut, have become very popular in India and Pakistan, especially among adolescents [44, 45]. These genotoxic agents might be acting synergistically along with the epigenetic machinery towards oral cancer pathogenesis. Considering the varied exposure to these populations, we compared the methylation profile of Indian patients with TCGA HNSC methylation data. Hypermethylated probes identified in OSCC tissues of Indian patients are almost same with that of the TCGA samples. However, we determined a set of hypomethylated probes that are specific to the Indian patients. Promoter of some of the carcinogen-metabolizing genes like *CYP8B1* and *GSTA3* was found to be hypomethylated, specifically, in the tumor tissues of OSCC patients in India (Additional file 4: Table S4). Gene ontology analysis showed enrichment of immune genes for the hypomethylated promoter in both common and unique clusters. These data reflects the cell-type composition of the tumor microenvironment and, in particular, lymphocyte infiltration in these tumors. A closer look at these genes revealed that the hypomethylation occurred at promoters of genes involved in T lymphocytes regulation. Genes encoding cytotoxic T cell markers, like the *CD8* and T cell activation markers, like *CD28*, *CD80*, *CD86*, *ZAP70*, *PI3* kinase or the *PTPN22* tyrosine phosphatase involved in T cell receptor signaling, and *CTLA4* involved in negatively regulating cytotoxic T cell signaling, are hypomethylated and overexpressed in the neoplastic environment. We considered whole tumor tissues for our DNA methylation analysis. These tissues are composed of mainly epithelial cells; however, cells from the surrounding stroma, including fibroblasts, extracellular matrix, and immune cells, are also present in the microenvironment [46]. Hypomethylation of immune genes in unique and common clusters are clear indication of infiltration of immune cells into tumors. Ingenuity pathway analysis with the unique differentially methylated promoters revealed enrichment of a distinct set of genes involved in immune response pathways. CTLA4 signaling in cytotoxic T lymphocytes is one of the most significant pathway enriched by these unique set of hypomethylated genes. These data indicated that the OSCC patients in India induce an anti-tumor T cell response, with mobilization of T lymphocytes in the tumor microenvironment. This is in accordance with the different oral habits present among the OSCC patients in India.

Our study has identified 162 uniquely hypomethylated probes that mapped to 134 genes, associated with OSCC patients in India. Survivability analysis showed that expression of 19 of these genes was significantly correlated with better disease prognosis. None of these genes have previously been reported in OSCC. Interestingly, nine (*CCR10*, *SLAMF1*, *TNFRSF4*, *APOBR*, *CLNK*, *CTLA4*, *FCRL6*, *P2RY14*, and *ZAP70*) out of these 17 genes are known to be involved in immune response pathways, and better disease prognosis was achieved with higher expression of these genes. Signaling-lymphocytic-activation-molecule-family1 (SLAMF1) is mainly involved in signal transduction for lymphocyte activation [47]. Lower expression of SLAMF1 was found to be associated with unfavorable prognosis in CLL [48]. Tumor necrosis factor receptor superfamily, member 4 (TNFRSF4) is a co-stimulatory molecule, and agonists of this molecule have been described to increase anti-tumor immunity through enhancing T cell response and suppressing T reg cells [49, 50]. TLR9 on plasmacytoid dendritic cells are involved in secretion of type I interferons that are involved in anti-tumor immunity [51]. Interaction of chemokine receptor 10 (CCR10) with its ligand causes T cell homing and metastasis in melanoma; higher expression of CCR10 was correlated with lower survival of glioblastoma patients [52]. ZAP-70 (zeta-chain (TCR)-associated protein kinase), involved in T cell activation, was however reported to show poor prognosis in B-CLL with higher expression [53]. Most importantly, we have observed all these genes to be regulated by hypomethylated regions in promoter that are unique among OSCC patients in India. Survivability analysis showed significant prognostic implications of expression profile of *CTLA4* gene involved in negative regulation of the immune system [54–56]. Higher expression of the early-stage patients was observed to be associated with better survival of the patients. However, this is contradictory to the reported functionality of these genes in immune suppression. Higher expression of these genes causing immunosuppression should have resulted in poor prognosis of the disease [57–60]. For example, CTLA4 is known to be expressed by T reg cells and activated T cells to prevent further T cell activation and thereby causing inhibition of the anti-tumor immune response [54]. Interestingly, we observed better survivability with higher *CTLA4* expression, only for the early-stage (stages I and II) OSCC patients. A humanized anti-CTLA4 monoclonal antibody (ipilimumab) was approved by the Food and Drug Administration (FDA) for metastatic and advanced melanoma. Clinical trials and combination trials are going on for other advance stage cancers including prostate, bladder, and lung cancer. Better survivability for the early-stage OSCC patients suggests that CTLA4 might be overexpressed on the surface of early-stage tumor cells, as have been observed on cancer cell lines as well as in non-small cell lung carcinoma [61, 62]. Binding of CTLA4 with its ligand on APCs triggered apoptosis of tumor cells further inhibited tumor progression. However, better understanding of the tumor microenvironment can lead to elucidation of the actual role of CTLA4 in OSCC prognosis.

## Conclusions

In summary, genome-wide DNA methylation profiling among OSCC patients in India thus revealed a set of differentially methylated regions, which were replicated in 60–90% patients in a separate cohort of OSCC patients. Comparison of the observed methylation pattern with TCGA HNSC methylation data revealed 94.6% similarities, indicating their important role in cancer development. Interestingly, we also observed 5.3% probes that were uniquely differentially methylated in Indian patients, which might be attributed to different oral habits observed in Indian patients. Further studies correlating methylation pattern with different oral habits might give insights into their role in cancer development. Gene ontology and IPA showed enrichment of differentially methylated promoters in immune regulation pathways including those mediated by CTLA4 and IL9 signaling. Our study thus highlights the finding of a distinct set of differentially methylated regions among Indian OSCC patients as well as a strong immune component that is regulated by methylation.

## Methods

### Patient selection and sample collection

After clinical inspection, patients with provisional diagnosis of oral squamous cell carcinoma (OSCC) were recruited for the study with their written consent. Sixty-four patients were included in the study after confirmation of well-differentiated squamous cell carcinoma from histopathological reports. The study was approved by the Institutional Ethics Committee for Human Research of Indian Statistical Institute, Kolkata, India. Tissues from the area of the lesion and adjoining clinically uninvolved area were collected using incisional and 3-mm punch biopsy, respectively for all 64 patients. A portion of the tissue samples were collected in RNA Later (Invitrogen) and stored at −80 °C until processing. Another portion was fixed in the formalin and used for histopathological evaluations.

Demographic information of the OSCC patients for both discovery and validation cohort are presented in Table 1 and Additional file 1: Table S1. Mean age of the patients of discovery set was 51.37 (SD ± 14.33) (range 40–90 years), while that of validation cohort was 53.35 (SD ± 12.35) (range 27–78 years). The patient samples of discovery and validation cohort had no significant difference of age (*P* value = 0.306), as calculated using the D’Agostino-Pearson omnibus normality test. There was no significant deviation in the distribution of males and females among OSCC patients (*P* value = 0.529) in both cohorts. Nearly 82% cases from the both cohorts had oral habits.

### Illumina Infinium HumanMethylation450K bead chip and data analysis

DNA was isolated from the lesion and adjacent uninvolved area using DNeasy Blood and Tissue Kit (Qiagen) following manufacturer’s protocol. The purity and concentration of DNA was estimated using Nanodrop 2000 (ThermoScietific). Approximately, 500 ng of genomic DNA from each sample was used for sodium bisulfite conversion using the EZ DNA methylation Gold Kit (Zymo Research, USA) following the manufacturer’s standard protocol. Genome-wide DNA methylation was assessed using the Illumina Infinium HumanMethylation450 BeadChip (Illumina Inc, USA) according to manufacturer’s instructions. Ten paired and one unpaired DNA samples were subjected to Infinium HumanMethylation450K bead chip analysis to interrogate 485,577 methylation sites per sample at single-nucleotide resolution. The array data (.IDAT files) was analyzed using RnBeads package in R for deriving the methylation level [63]. The methylation status of all the probes was denoted as β value, which is the ratio of the methylated probe intensity to the overall probe intensity (sum of methylated and unmethylated probe intensities plus constant α, where α = 100). CpG sites having |Δβ| ≥0.20 (in OSCC vs adjacent normal) and adjusted *P* value ≤0.05 was considered as differentially methylated site. A CpG was considered hypermethylated if Δβ ≥0.20 or hypomethylated if Δβ ≤−0.20. Average β value of promoters and CpG islands were compared between disease and normal. Promoters and CGIs with |Δβ| ≥0.20 and adjusted *P* value ≤0.05 were considered for further analysis. Heatmaps were generated using gplots package in R. The data has been submitted to the Gene Expression Omnibus (GEO) with accession number GSE87053.

### HPV determination by PCR

Presence of integrated HPV sequences was detected from DNA isolated from diseased tissue by PCR with primers specific for the conserved L1 region of viral genome using the method as described previously [64].

### Comparison with TCGA methylation data

The methylation data of our study were compared with a publically available, open access methylation dataset of HNSC from The Cancer Genome Atlas (TCGA) (https://tcga-data.nci.nih.gov/docs/publications/tcga/?). In order to ensure equivalent comparisons, TCGA samples with malignancies at the larynx, alveolar ridge, oropharynx, hypopharynx, and tonsil were not included in this study (Additional file 3: Table S2). RnBeads package was used for analysis of TCGA samples. The differentially methylated probes, promoters and CpG islands observed in our samples were compared with the TCGA samples.

### Identification of common and unique probes

The total hypomethylated probes were categorized into two categories viz common and unique. All stages (S1–S4) of TCGA data were compared with OSCC disease samples. The unique probes were significantly different (adjusted *P* value ≤0.05) from each stages of TCGA samples. Those DMPs that were similar (i.e., not significantly different) to any stages of the TCGA samples were considered as common hypo- or hypermethylated probes.

### Bisulfite sequencing PCR and quantitative real-time PCR for methylation analysis

The bisulfite sequencing PCR (BSP) were carried out for 14 promoters by bisulfite conversion-specific primers (Additional file 5: Table S5) designed using MethPrimer [65]. The BSP products were analyzed on 1% agarose gel. A part of BSP products were then used for the quantitative real-time methylation-specific PCR (qMSP). The qMSP reactions were carried out using methylation-specific primers (Additional file 5: Table S5) with FastStart Universal SYBR Green Master Mix (Rox) (Roche, Switzerland) in a 7900HT Fast Real-Time PCR System Instrument (ABI, USA).

### BSP cloning and sequencing

The BSP products were then selected for cloning and sequencing. Another part of the BSP products was purified with the MiniElute Gel extraction Kit (Qiagen Inc., USA). About 165 ng of the purified BSP product was ligated in the TA vector (PTZ57R/T) using T4 DNA Ligase (Takara Bio Inc., Japan). The ligated product were then transformed into *E.coli*DH5α cells and plated on LA-Ampicillin plates. The probable positive clones were verified by colony PCR using universal M13 forward and reverse primer and under standard conditions. The positive clones were then sequenced in 3100 Genetic Analyzer (ABI, USA). The status of methylation of each clone were analyzed from the chromatogram thus obtained, and the percentage methylation was calculated.

### Gene expression study

Twenty paired tissue samples were selected for expression analysis of the identified genes with differentially methylated promoters. Biopsy samples stored in RNA Later Solution (Invitrogen, USA) at −80 °C were used. Tissue samples were snap frozen in liquid nitrogen and grinded using mortar and pestle. Total RNA was extracted using High Pure RNA Tissue Kit (Roche). Quality of the eluted RNA was checked in Nanodrop spectrophotometer. Samples with low RNA yield or impure RNA was not considered for further study. One microgram of total RNA was used for complementary DNA (cDNA) synthesis using RevertAid First Strand cDNA Synthesis Kit (Thermo Scientific). The product was subsequently diluted, and around 10 ng was finally used for each reaction. Transcripts were quantified using specific gene expression primers (Additional file 6: Table S6) in 7900HT Fast Real-Time PCR system (Applied Biosystems) using FastStart Universal SYBR Green Master (Rox) (Roche). All values were normalized to the expression of the housekeeping gene ribonuclease P (RPP30).

### Survival analysis

To determine the clinical significance of the unique hypomethylated genes, we analyzed methylation and expression values of all 134 genes with the clinical follow up data available at TCGA. Survival analysis for the gene expression data were performed using OncoLnc [66]. Top and bottom 25 percentile of expression values were considered as high and low groups, respectively. Survival analyses of the unique DMPs were done using the Kaplan-Meier survival analysis in SPSS. Mantel-Cox log-rank *P* value ≤0.05 was considered as significant. The patients were sorted based on their β values for each unique DMP. Low and high groups are generated from the top and bottom 25 percentile patients.

## Additional files

Additional file 1: Table S1. Clinical and demographic charecteristics of the discovery and validation cohort samples. (XLSX 14 kb)
Additional file 2: Figure S1. Histopathogical images of mis-clustered samples. **Figure S2.** Genomic distribution of DMPs. **Figure S3.** Hierarchical clustering of average methylation. **Figure S4.** Validation of HumanMethylation450K bead chip data. **Figure S5.** Expression profile of genes with hypermethylated promoters. **Figure S6.** Expression profile of genes with hypomethylated promoters. **Figure S7.** Comparison of methylation pattern in adjacent normal, OSCC, and different stages of TCGA sample. **Figure S8.** Comparison of common hypomethylated probes. **Figure S9.** Comparison of unique hypomethylated probes. **Figure S10.** Gene ontology analysis of DMPs. **Figure S11.** Clinical significance of immune genes associated with uniquely hypomethylated probes. **Figure S12.** Clinical significance of non-immune genes associated with uniquely hypomethylated probes. (PDF 2377 kb)
Additional file 3: Table S2. Clinical and demographic charecteristics of the TCGA samples included in this study. **Table S3.** Hazard ratio estimated in cox model based on death due to oral cancer by age, stage and node involvement. (XLSX 54 kb)
Additional file 4: Table S4. Spreadsheet showing list of all unique differentially methylated probes. (XLSX 23 kb)
Additional file 5: Table S5. Primer sequences used for BSP and qMSP studies. (DOCX 16 kb)
Additional file 6: Table S6. Primer sequences used for gene expression study. (DOCX 13 kb)
