# Supplementary material for: Genome-wide DNA methylation profile identified a unique set of differentially methylated immune genes in oral squamous cell carcinoma patients in India
Source: Clin Epigenetics. 2017 Feb 3;9:13. doi: 10.1186/s13148-017-0314-x (PMC5292006; doi:10.1186/s13148-017-0314-x)

Additional File 2

Figure S1. Histopathogical images of mis-clustered samples. A) Case no.33: Severely dysplastic epithelium invading into underlying connective tissue with multiple keratin pearls highlighted in the square box. Arrow indicates the breakdown of the basement membrane and invasion. **B)** Case no.33: H&E stained photomicrograph highlights the square box portion of figure (A). It shows formation of multiple keratin pearls in the underlying connective tissue. **C)** Case no.33: H&E stained photomicrograph showing adjacent clinically and histologically normal stratified squamous epithelium. **D)** Case No.37: Photomicrograph of adjacent clinically normal site showing featurs of hyperplastic stratified squamous epithelium.
E) Case No.37: Neoplastic tumor islands in case of well differentiated SCC with individual cell keratinisation (dyskeratosis). **F)** Case no. 84: Multiple tumor islands invading into underlying connective tissue with discrete Keratin pearl formation. **G)** Tissue from adjacent anatomic location of lesion. Clinically normal but H&E section showing mild features of dysplasia with Arrows indicating abnormal mitotic figures and increased mitoses in suprabasal layers of stratified squamous epithelium.

Figure S2. Genomic distribution of DMPs. Distribution of **A)** 4,63,430 probes profiled in HumanMethylation450K bead chip and their localization at the CGIs and Non-CGIs, **B)** all differentially methylated probes (DMPs), Hyper- and Hypomethylated Probes and their enrichment on CGIs and Non CGIs. **C)** Genomic localisation of Hypermethylated (5,670) probes in promoters, exons, introns, repeats and other regions. **D)** Genomic localisation of Hypomethylated probes (16,140) in promoters, exons, introns, repeats and other regions.

Figure S3. Hierarchical clustering of average methylation. Heatmap showing hierarchical clustering of average β values for the top differentially methylated **A)** promoters and **B)** CpG islands between OSCC and adjacent normal tissues.

Figure S4. Validation of HumanMethylation450K bead chip data. Clonal validation by sequencing bisulfite converted products of **A)** *HLA-DPB1*, **B)** *LDLRAD4* promoters and **C)** last exon of *LHX1*.

Figure S5. Expression Profile of Genes with Hypermethylated Promoters. Gene expression profile of **A)** *ZNF154*, **B)** *CTDSP1*, **C)** *ZNF577*, **D)** *ZSCAN31*, **E)** *LDLRAD4* and **F)** *HLA-DPB1* with hypermethylated promoters. Differential expression was observed between normal and OSCC tissues, and promoter methylation status was inversely correlated with gene expression. P-value ≤ 0.05 was considered significant.

Figure S6. Expression Profile of Genes with Hypomethylated Promoters. Gene expression profile of **A)** *RUNX1*, **B)** *IL6*, **C)** *CD28*, **D)** *CD80*, **E)** *TLR1* and **F)** *TNFα* with hypomethylated promoters. Differential expression was observed between normal and OSCC tissue samples, and promoter methylation status was inversely correlated with gene expression. P-value ≤ 0.05 was considered significant..

Figure S7. Comparison of methylation pattern of common hypermethylated probes in adjacent normal, OSCC and different stages of TCGA sample. Box plots showing

average β values for the DMPs at promoters of **A)** *HLA-DPB1,* **B)** *LXN,* **C)** *ZNF577,* **D)** *ZNF154,* **E)** *LDLRAD4,* **F)** *CTDSP1* and **G)** *LHX1*. Methylation profile of the gene promoters in disease tissue of Indian patients (Disease) was significantly different from their normal counterpart (Normal), but not significantly different from atleast one of disease stages S1, S2, S3 and/or S4A of TCGA samples. P-value ≤ 0.05 was considered significant.

Figure S8. Comparison of common hypomethylated probes. Box plots showing average β values for the DMPs at promoters of **A)** *RUNX1*, **B)** *CD28*, **C)** *PTPN22* and **D)** *TLR1*. Methylation profile of the gene promoters in disease tissue of Indian patients (Disease) was significantly different from their adjacent normal tissue (Normal), but not significantly different from atleast one of disease stages S1, S2, S3 and/or S4A of TCGA samples. P-value ≤ 0.05 was considered significant.

Figure S9. Comparison of unique hypomethylated probes. Box plots showing avergae β values for the DMPs at promoters of **A)** *TNF,* **B)** *IL27,* **C)** *TLR9,* **D)** *TNFRSF4,* **E)** *IL9* and **F)** *CD22*. Methylation profile of the gene promoters in disease tissue of Indian patients (Disease) was significantly different from: their adjacent normal tissue (Normal), as well as from all of disease stages S1, S2, S3 and S4A of TCGA samples. P-value ≤ 0.05 was considered significant.

Figure S10. Gene ontology analysis of DMPs. Bar plots show $-\log_{10}$(P-value) versus enriched biological proceeses of **A)** Common hypermethylated probes **B)** Common Hypomethylated probes and **C)** Unique hypomethylated probes.

Figure S11. Clinical significance of immune genes associated with uniquely hypomethylated probes. Kaplan-Meier plots with expression values of uniquely hypomethylated genes that involved in immune response pathways: **A)** *CCR10*, **B)** *SLAMF1*, **C)** *TNFRSF4*, **D)** *APOBR*, **E)** *CLNK*, **F)** *FCRL6*, **G)** *P2RY14*, **H)** *TLR9* and **I)** *ZAP70.* Plots were generated with expression levels and survival information obtained from TCGA data.

Figure S12. Clinical significance of non-immune genes associated with uniquely hypomethylated probes. Kaplan-Meier plots for uniquely hypomethylated genes that were significantly associated with disease prognosis but not related to immune response pathways: **A)** *SLC26A1*, **B)** *CACNA2D3*, **C)** *BSFP2*, **D)** *MFSD7*, **E)** *MTHFD1L* and **F)** *TKT*. **G**) Kaplan-Meier plot of *CTLA4* expression with Late Stage patients (Stage 3 and 4) of TCGA HNSC samples.

Supplementary Figure S1

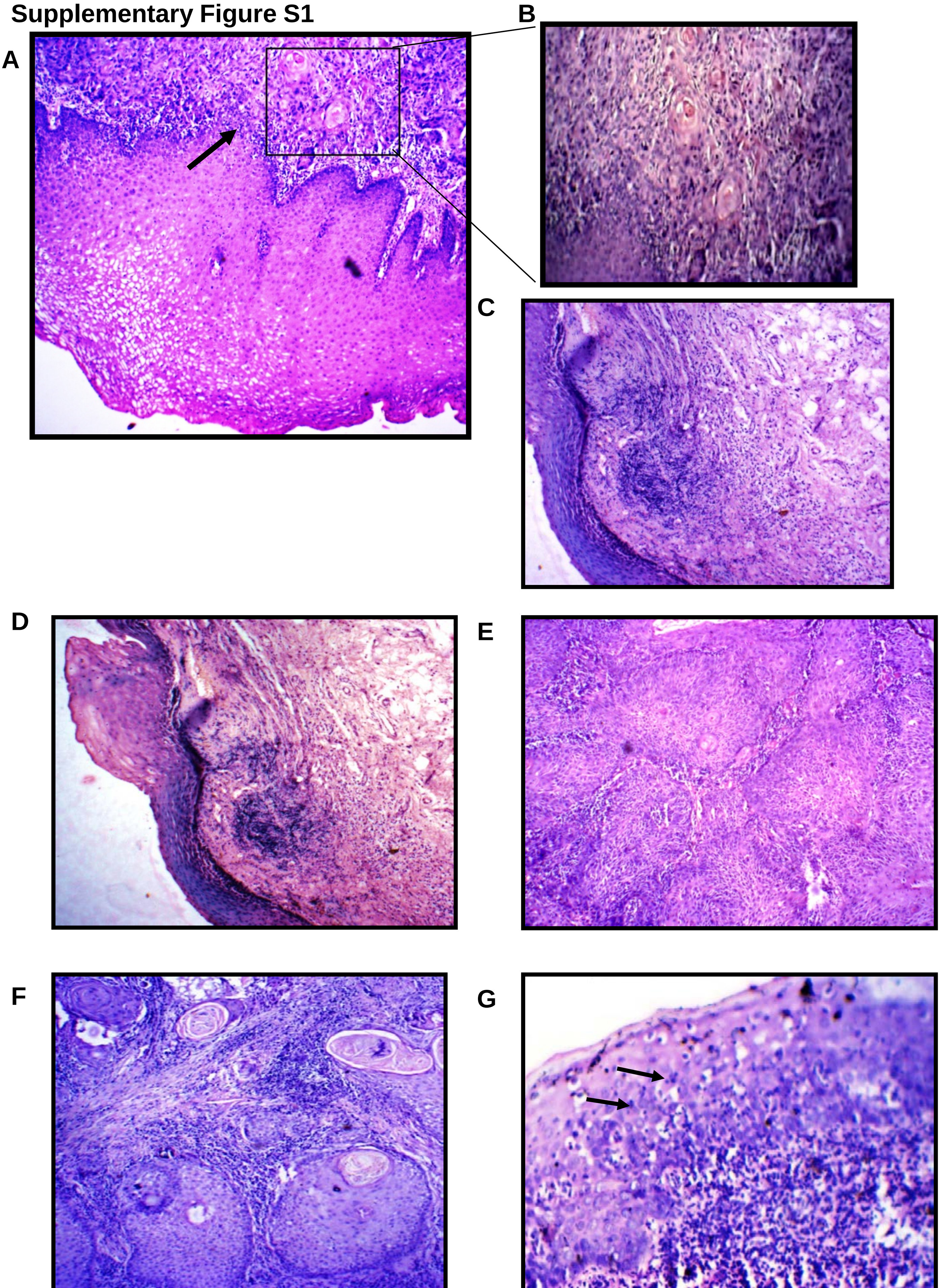

Supplementary Figure S2

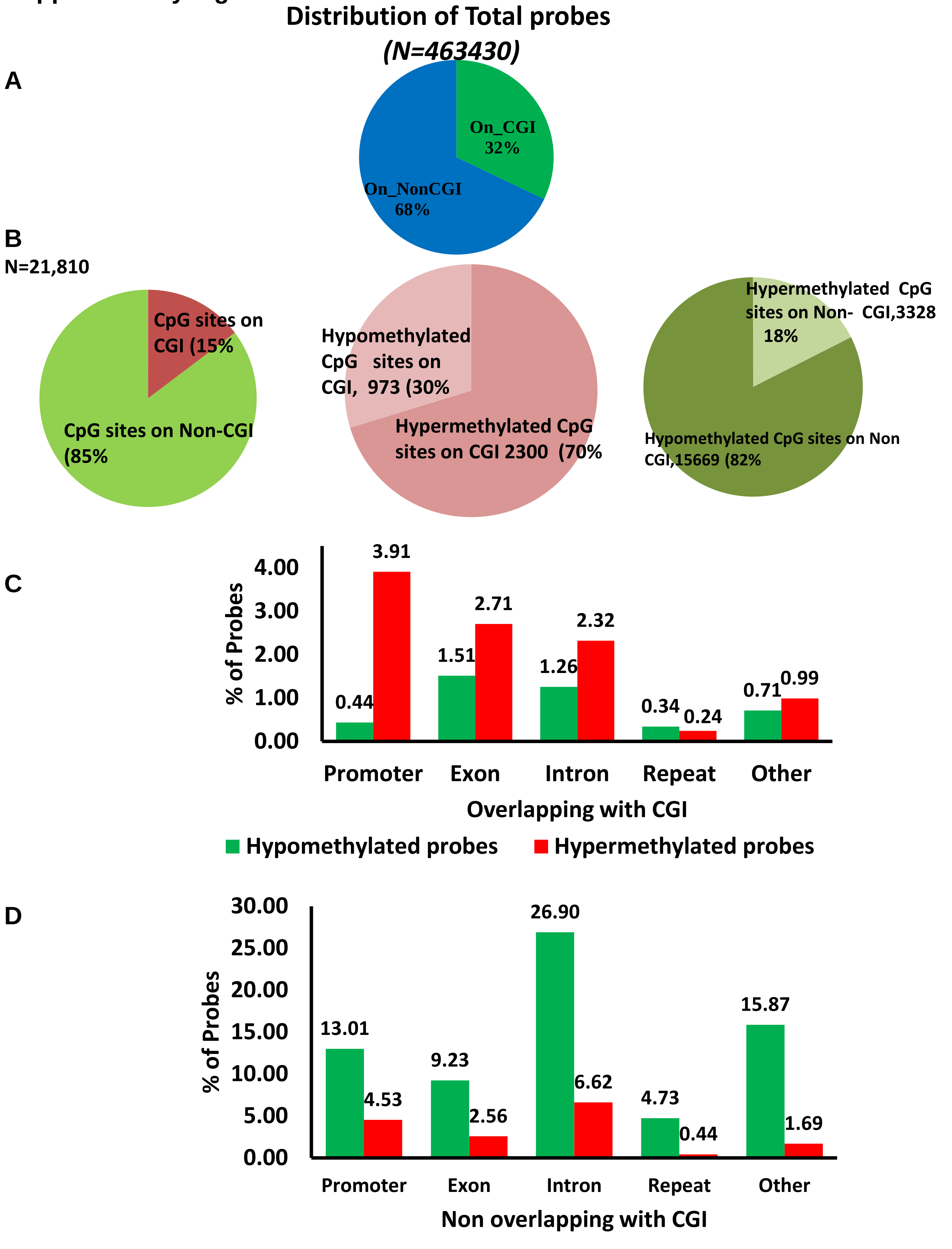

Supplementary Figure S3

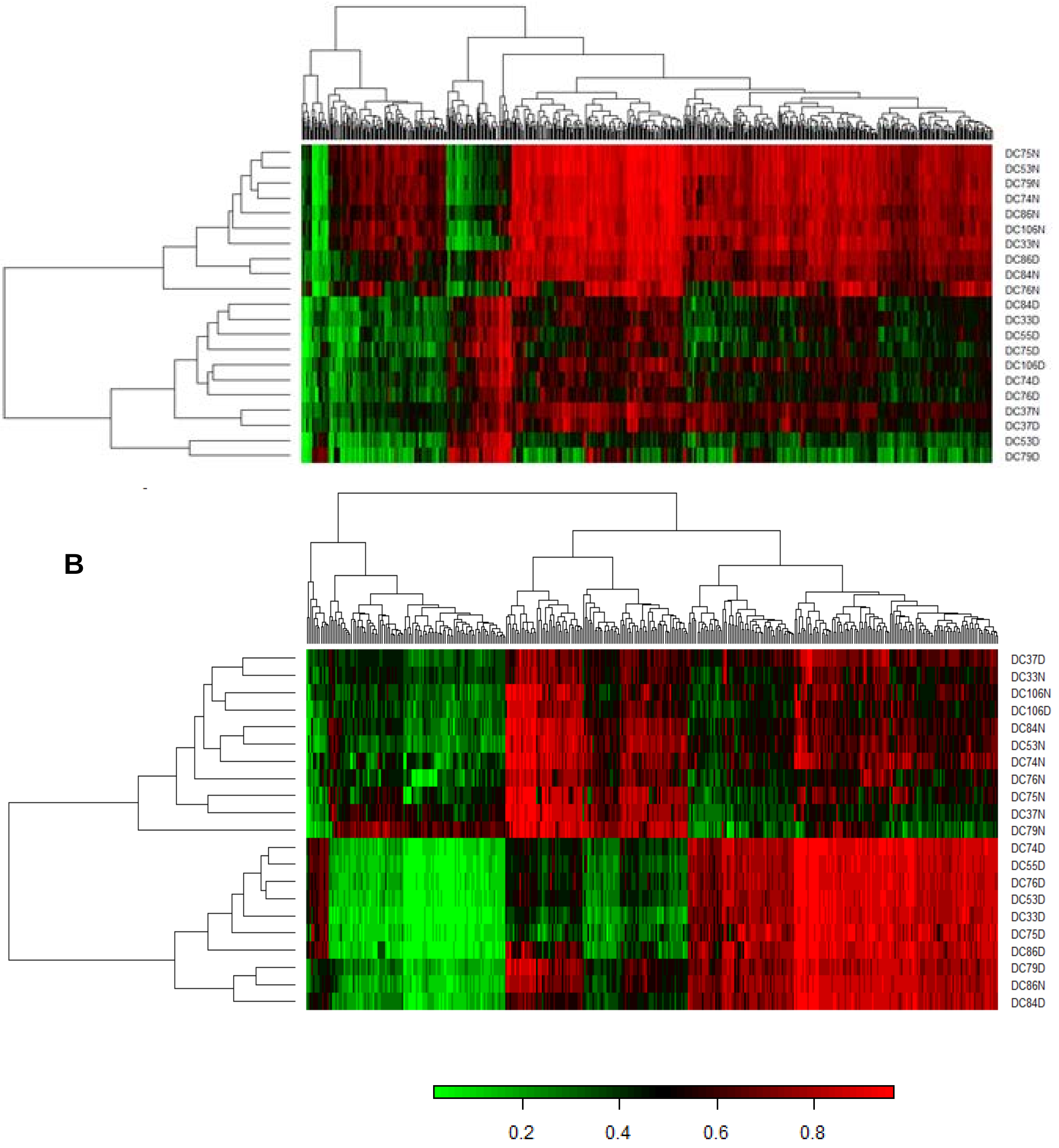

Supplementary Figure S4

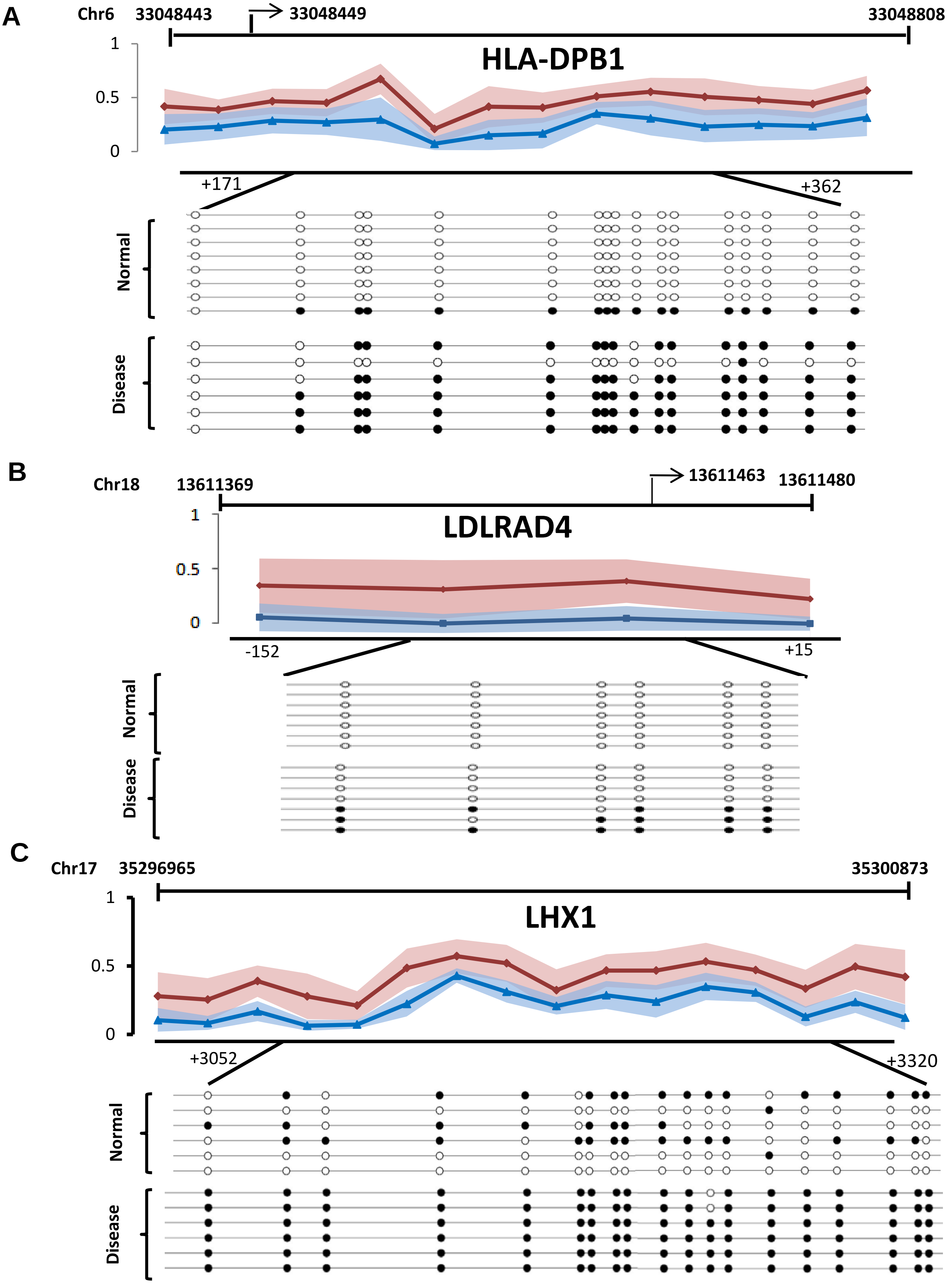

Supplementary Figure S5

Expression Profile of Genes with Hypermethylated Promoters

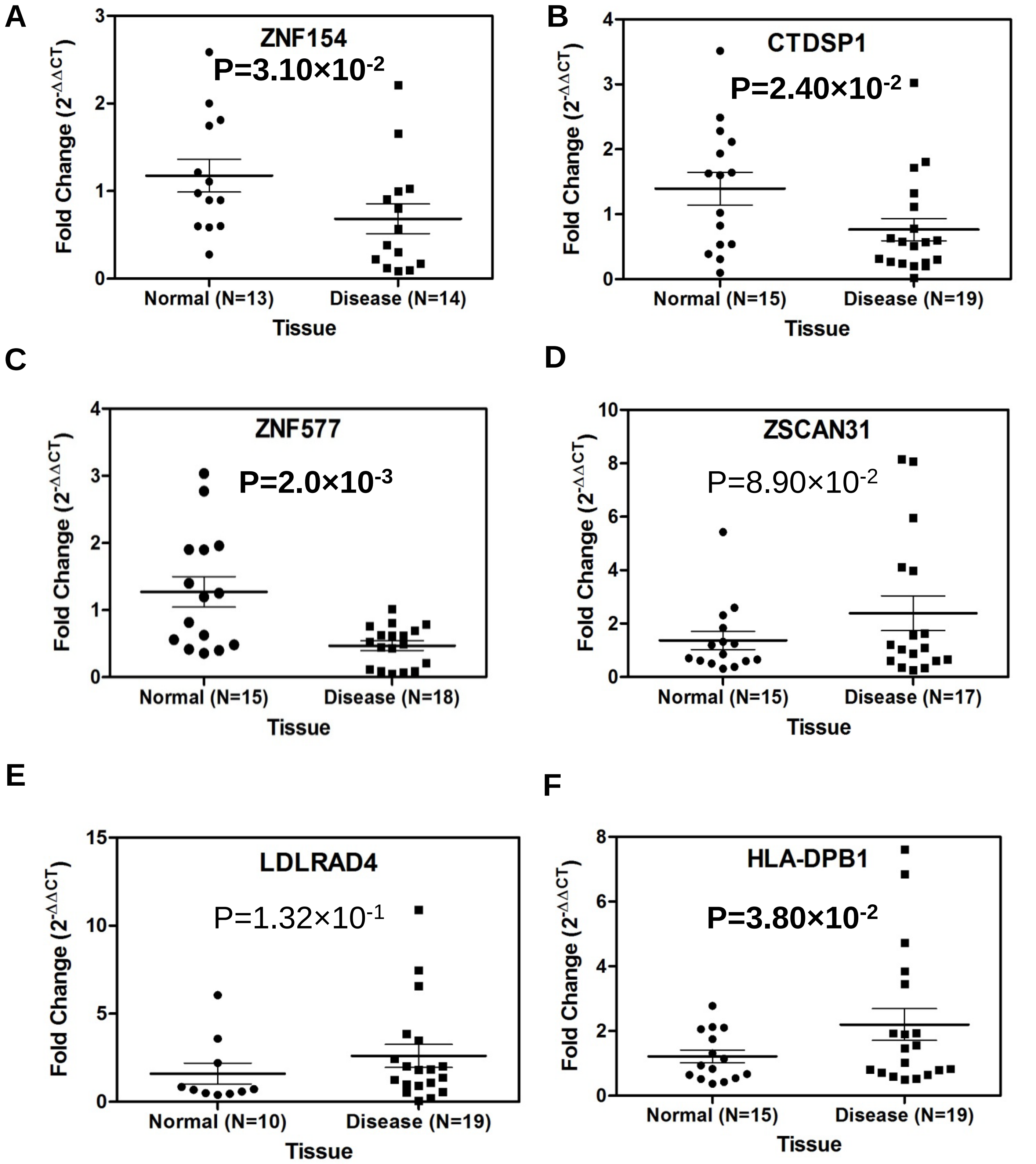

Supplementary Figure S6

Expression Profile of Genes with Hypomethylated Promoters

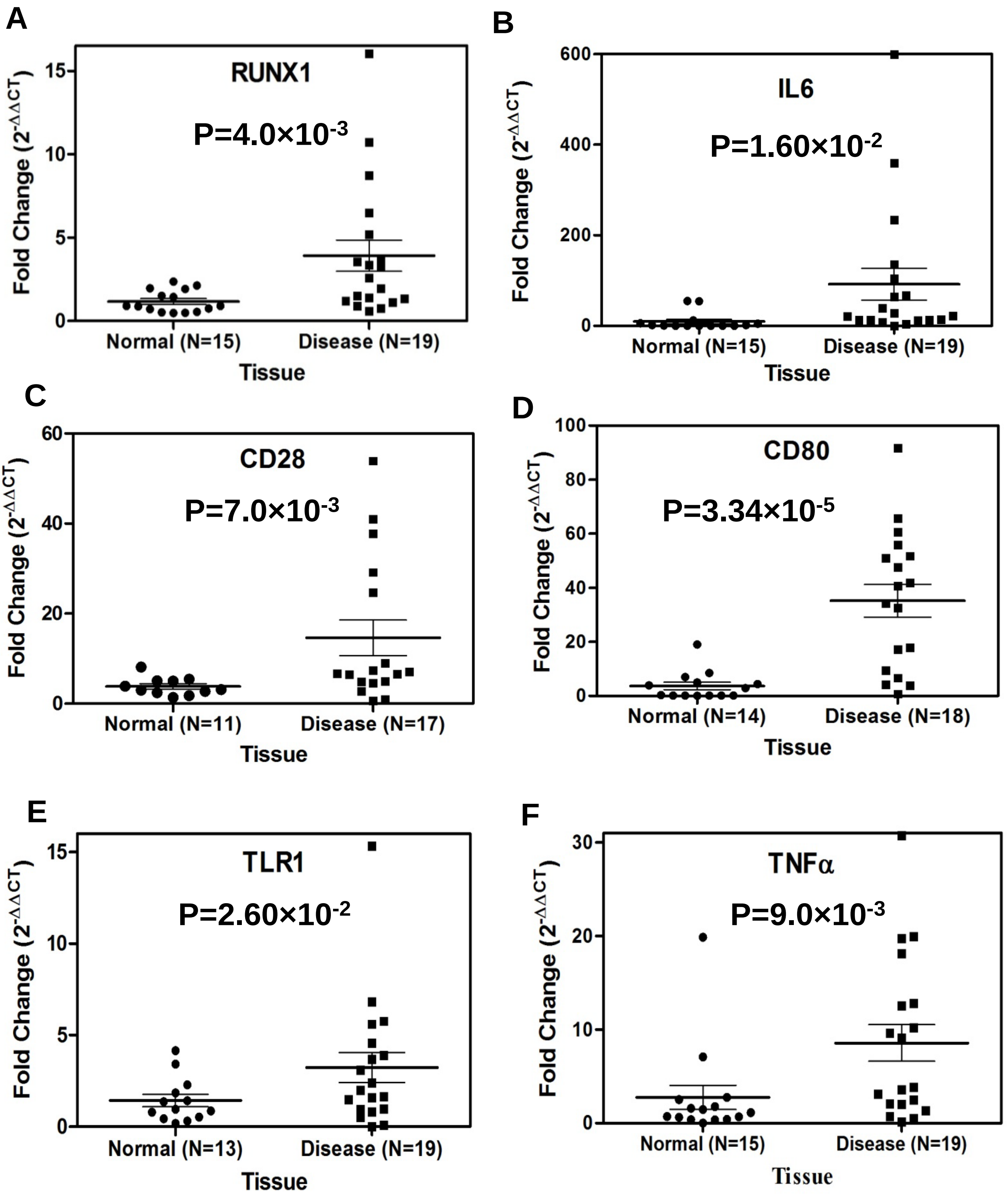

Supplementary Figure S7

Common Hypermethylated Probes

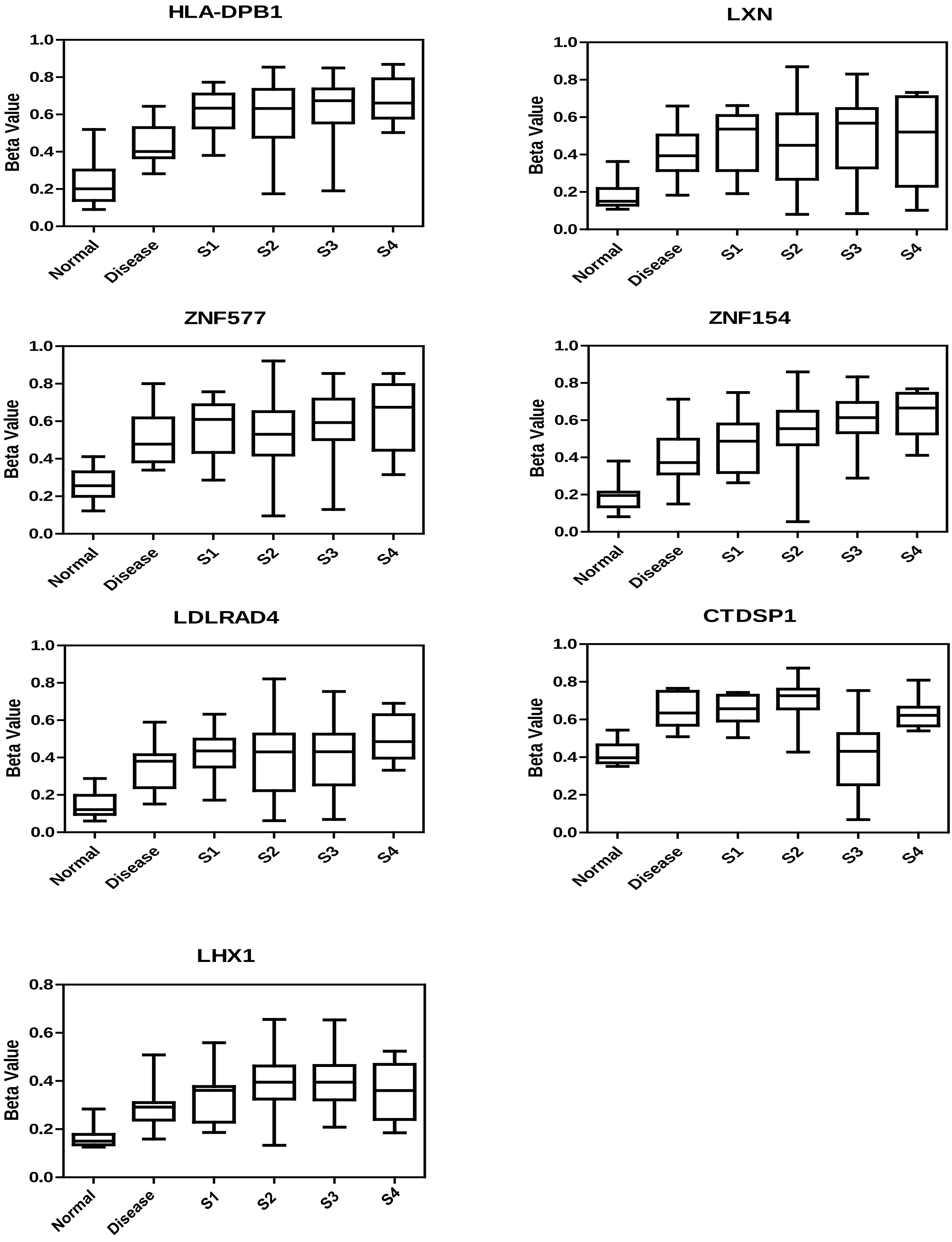

Supplementary Figure S8

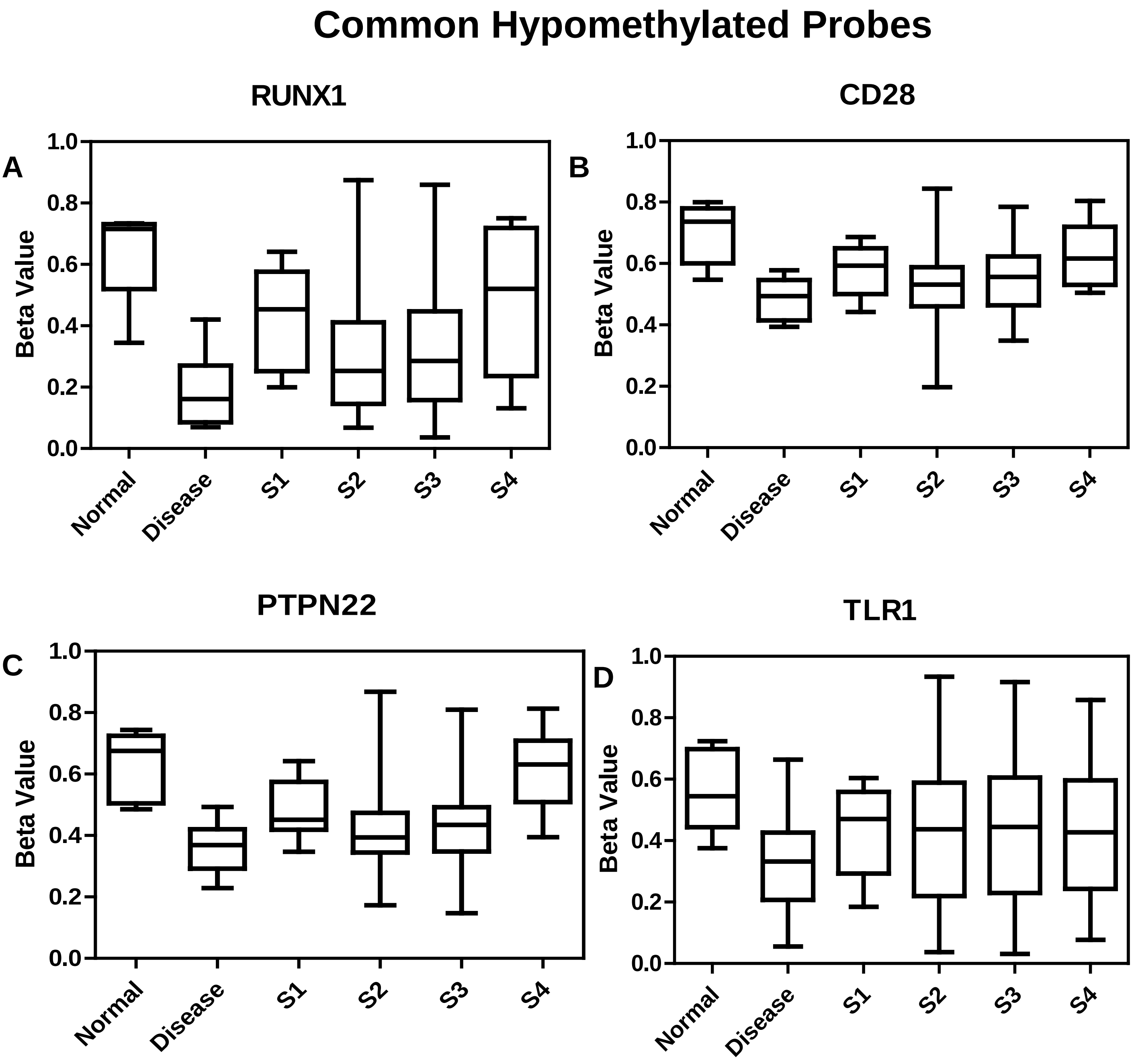

Supplementary Figure S9

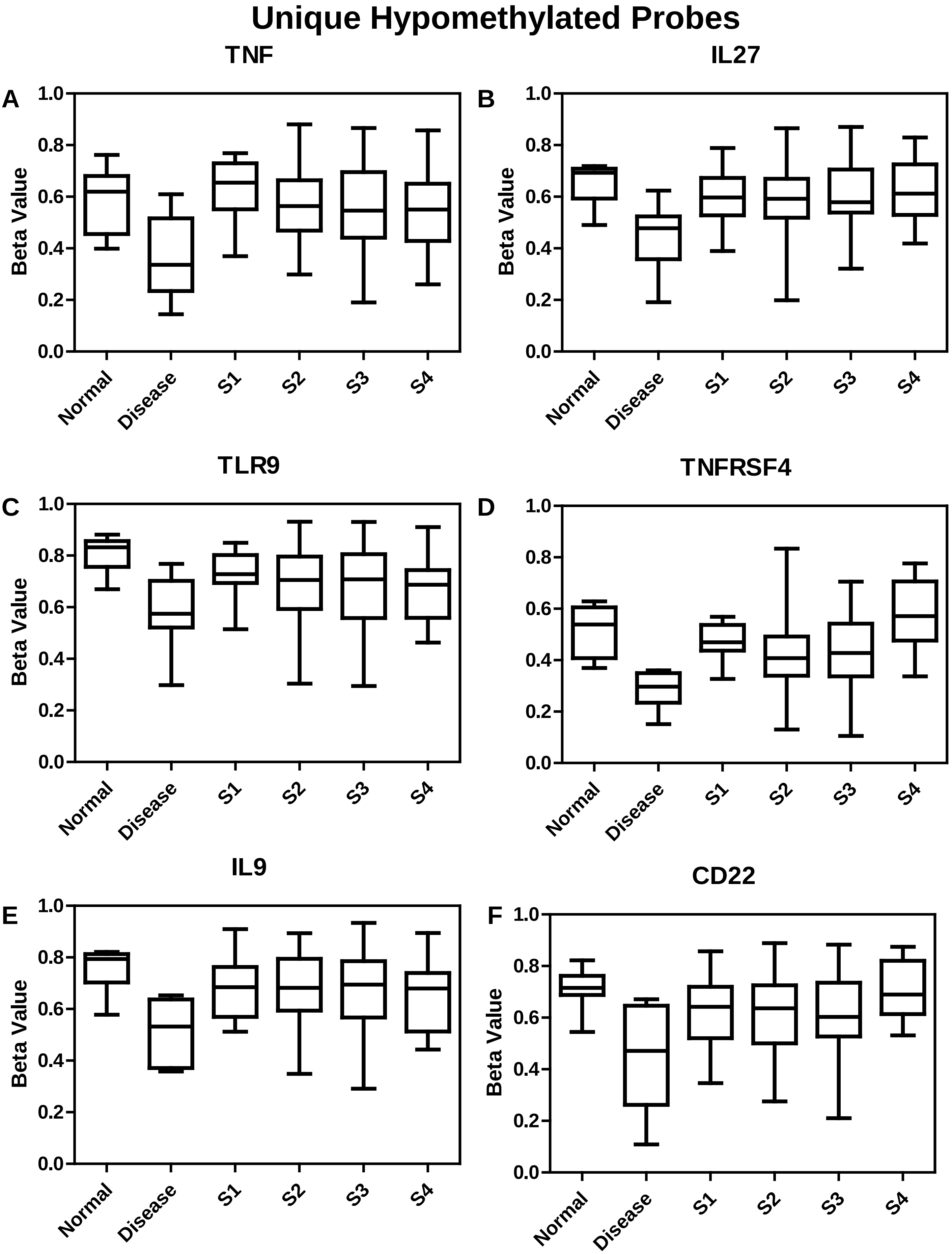

SupplementaryFigureS10

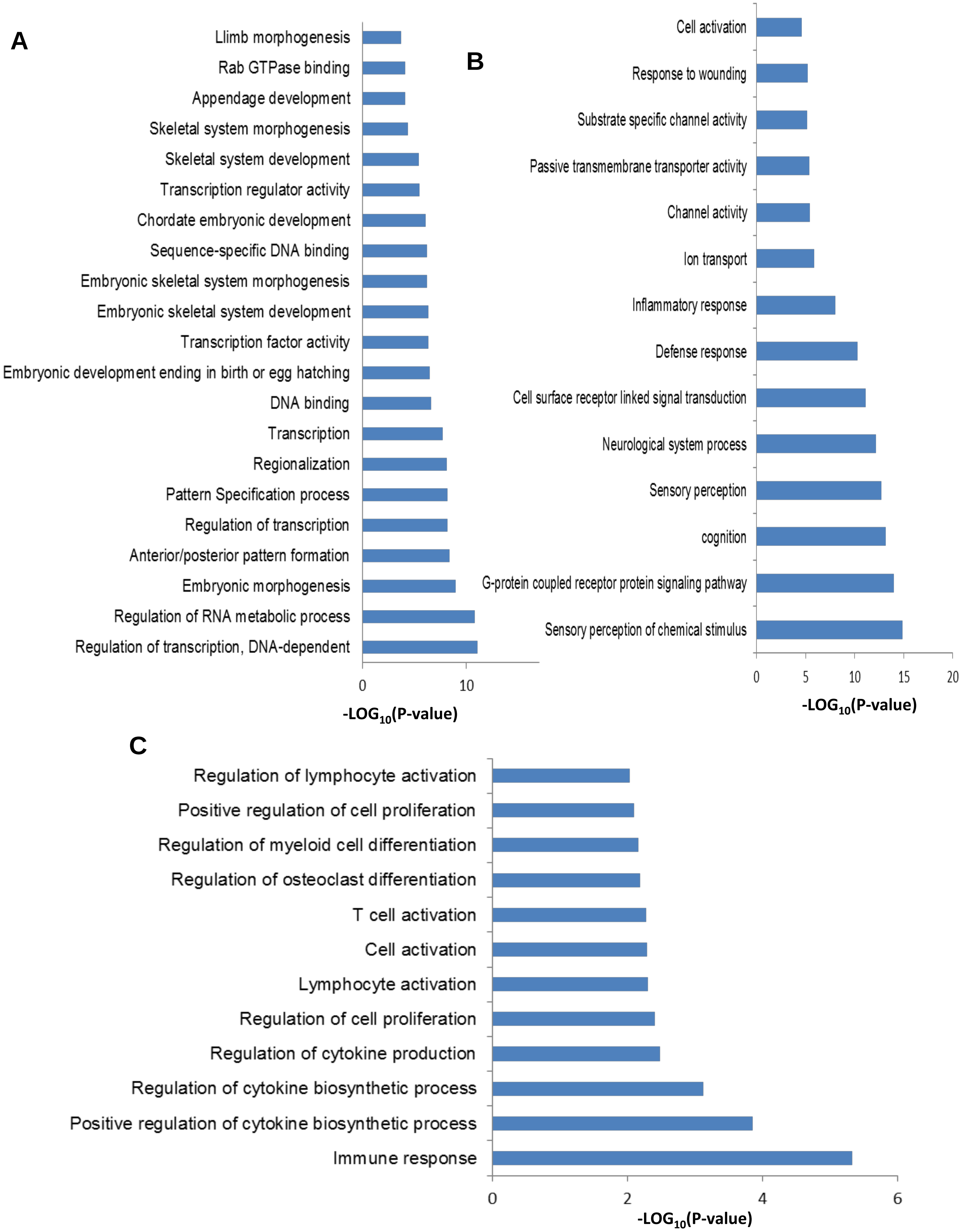

Supplementary Figure S11 Kaplan Meier Plots for uniquely hypomethylated genes related to immune response pathways

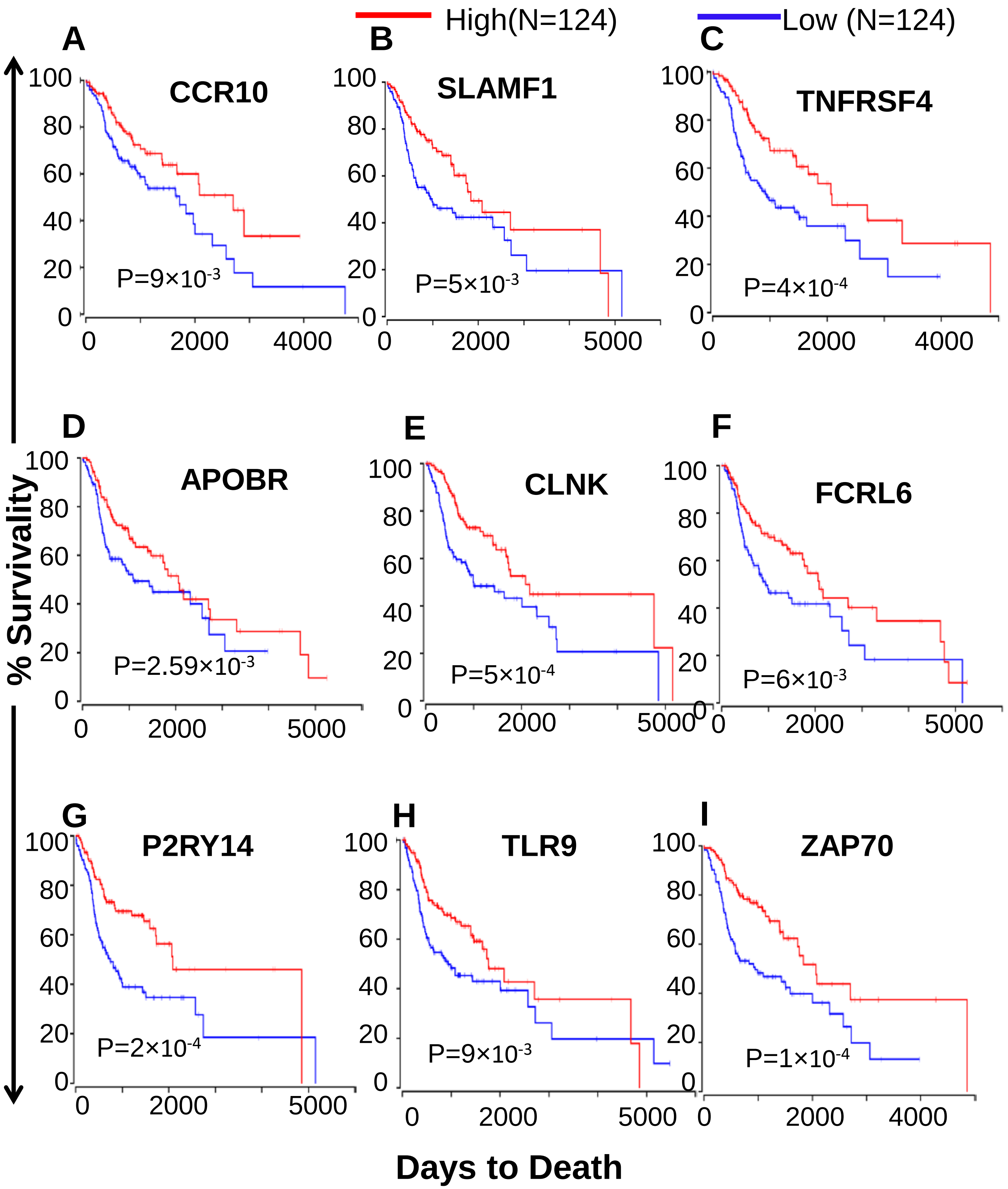

Supplementary Figure S12 Kaplan Meier Plots for other uniquely hypomethylated

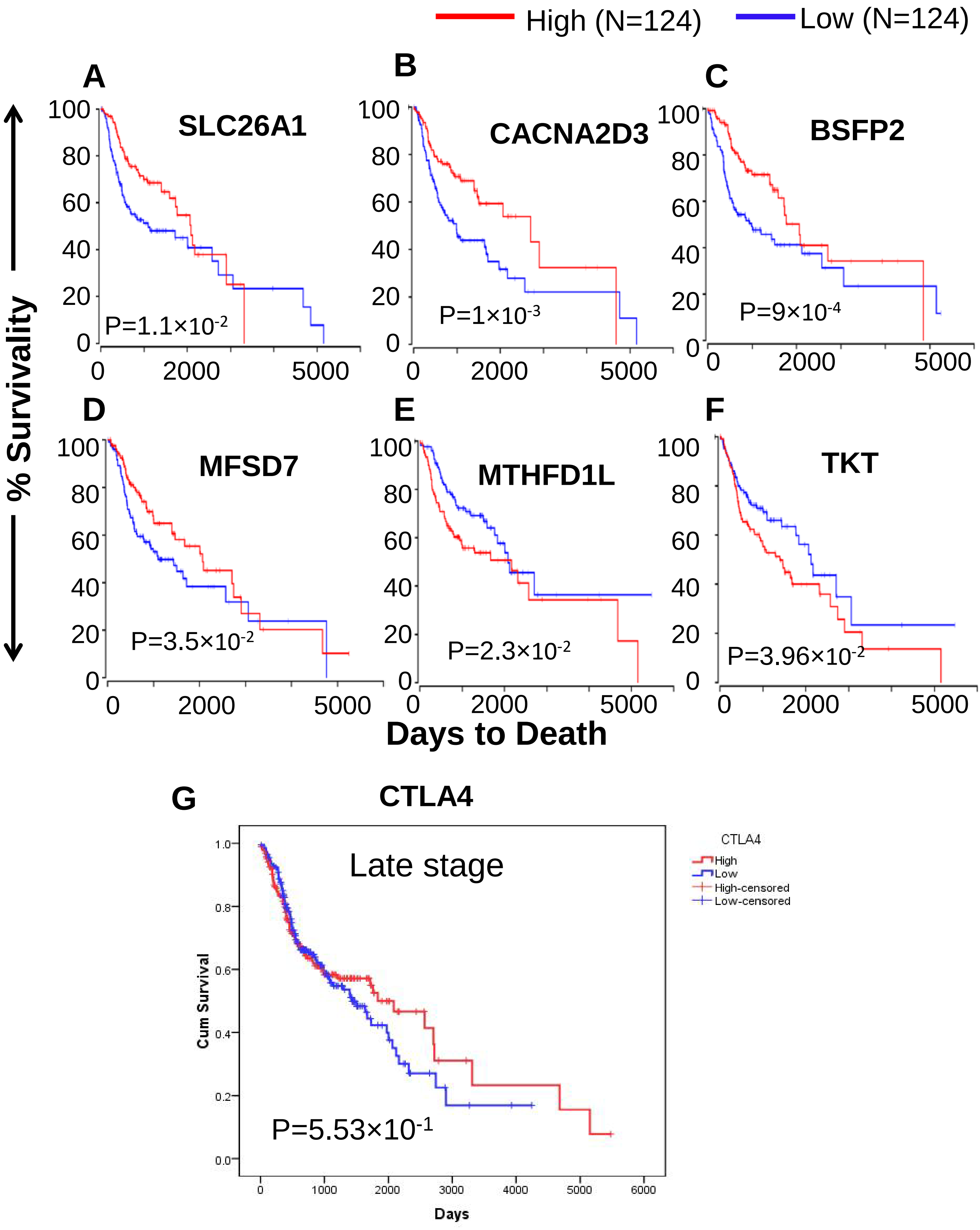

Supplement: Additional file 2: Figure S1. — Histopathogical images of mis-clustered samples. Figure S2. Genomic distribution of DMPs. Figure S3. Hierarchical clustering of average methylation. Figure S4. Validation of HumanMethylation450K bead chip data. Figure S5. Expression profile of genes with hypermethylated promoters. Figure S6. Expression profile of genes with hypomethylated promoters. Figure S7. Comparison of methylation pattern in adjacent normal, OSCC, and different stages of TCGA sample. Figure S8. Comparison of common hypomethylated probes. Figure S9. Comparison of unique hypomethylated probes. Figure S10. Gene ontology analysis of DMPs. Figure S11. Clinical significance of immune genes associated with uniquely hypomethylated probes. Figure S12. Clinical significance of non-immune genes associated with uniquely hypomethylated probes. (PDF 2377 kb) [file 13148_2017_314_MOESM2_ESM.pdf]
