# Supplementary material for: Genome-wide DNA methylation profile identified a unique set of differentially methylated immune genes in oral squamous cell carcinoma patients in India
Source: Clin Epigenetics. 2017 Feb 3;9:13. doi: 10.1186/s13148-017-0314-x (PMC5292006; doi:10.1186/s13148-017-0314-x)
Supplement: Additional file 5: Table S5. — Primer sequences used for BSP and qMSP studies. (DOCX 16 kb) [file 13148_2017_314_MOESM5_ESM.docx]

**Table S2: Primer sequences used for BSP and qMSP studies**

| Gene Promoter | Primer Name | Sequence |
| --- | --- | --- |
| ZNF577 | ZNF577_BSP_F | TTTTTGAAATATTTTTAGAAGTTTA |
|  | ZNF577_BSP_R | TTAAAAATAACTACTCCCAACAAAC |
|  | ZNF577_MSP_M_F | GATTTGTTTTTATTGTTGGAGGC |
|  | ZNF577_MSP_M_R | AAAACGACTAATACCGTATTCCGTA |
|  | ZNF577_MSP_U_F | ATTTGTTTTTATTGTTGGAGGTGA |
|  | ZNF577_MSP_U_R | AAAAACAACTAATACCATATTCCATA |
| HLA DPB1 | HLA_DPB1_BSP_F | GTAGAGAATTATTTTTTTTAGGGA |
|  | HLA_DPB1_BSP_R | ATCCTTCTAACTATTCCAATACTCC |
|  | HLA_DPB1_MSP_M_F | AGAGAATTATTTTTTTTAGGGACGG |
|  | HLA_DPB1_MSP_M_R | TCCTTCTAACTATTCCAATACTCCG |
|  | HLA_DPB1_MSP_U_F | AGAGAATTATTTTTTTTAGGGATGG |
|  | HLA_DPB1_BSP_U_R | TTCTAACTATTCCAATACTCCACA |
| LHX1 | LHX1_BSP_F | TAATTTTTGGTTGGTTAGGTGTAAG |
|  | LHX1_BSP_R | TCTCCAACTACTTAACTTTAATAATAATAC |
|  | LHX1_MSP_M_F | CGGGTAGTGATTTTAGTTTGTTTTC |
|  | LHX1_MSP_M_R | CAAATTCTAATCGTCATTCTCGTT |
|  | LHX1_MSP_U_F | TGGGTAGTGATTTTAGTTTGTTTTTG |
|  | LHX1_MSP_U_R | CCCAAATTCTAATCATCATTCTCAT |
| MYH14 | MYH14_BSP_F | GTTTGGGAGGGTGTTTGGTATA |
|  | MYH14_BSP_R | AAAACCCAAAAAACCAAAATAAATAA |
|  | MYH14_MSP_M_F | TGTTTTTTGTTTTTTTTGTTCGTC |
|  | MYH14_MSP_M_R | AAATAAATAAACCGCTTTATACGCA |
|  | MYH14_MSP_U_F | GTTTTTTGTTTTTTTTGTTTGTTG |
|  | MYH14_MSP_U_R | AAATAAATAAACCACTTTATACACA |
| ZSCAN31 | ZSCAN31_BSP_F | GGTTGTTGTTTGGTGATTTTTTTAT |
|  | ZSCAN31_BSP_R | ACCAAATTCCTAACTAAAACTCTTC |
|  | ZSCAN31_MSP_M_F | TAAAAATTTTAGTTTTATTGGGTGC |
|  | ZSCAN31_MSP_M_R | CCGTAAAATTACCTCCTATTACTCG |
|  | ZSCAN31_MSP_U_F | AAAATTTTAGTTTTATTGGGTGTGA |
|  | ZSCAN31_MSP_U_R | CCATAAAATTACCTCCTATTACTCACT |
| LDLRAD4 | LDLRA4_BSP_F | GGAAAAATAAAGGTTATTTTGGGTT |
|  | LDLRA4_BSP_R | ACCCCCAAACTATATAAAAAAAATTC |
|  | LDLRA4_MSP_M_F | AAAATAAAGGTTATTTTGGGTTTTC |
|  | LDLRA4_MSP_M_R | TTCAAAACAAACTACGAATCTATCG |
|  | LDLRA4_MSP_U_F | ATAAAGGTTATTTTGGGTTTTTGG |
|  | LDLRA4_MSP_U_R | CAAAACAAACTACAAATCTATCACA |
| LXN | LXN_BSP_F | TGGTTTGTTTGATTTTTTGTATTTTAA |
|  | LXN_BSP_R | AACCTAAACCCAAACAACAACTTC |
|  | LXN_MSP_M_F | TTTTTGTATTTTAAATATTTTGTGCG |
|  | LXN_MSP_M_R | GACGCTCTACTACTAATCCCGTT |
|  | LXN_MSP_U_F | TTTGTATTTTAAATATTTTGTGTGG |
|  | LXN_MSP_U_R | ACAACACTCTACTACTAATCCCATT |
| PIWIL1 | PIWIL1_BSP_F | TGGTTGTTGATTTTGTTTTTATGAG |
|  | PIWIL1_BSP_R | CAAAAATTCTCAACCTTCACCTAC |
|  | PIWIL1_MSP_M_F | TATGGAGGGTATTTAGTTCGGC |
|  | PIWIL1_MSP_M_R | AAAAATTCTCAACCTTCACCTACG |
|  | PIWIL1_MSP_U_F | TTATGGAGGGTATTTAGTTTGGTGT |
|  | PIWIL1_MSP_U_R | AAAAATTCTCAACCTTCACCTACAA |
| MFAP2 | MFAP2_BSP_F | ATTTGAAAAATTTTTTTTGTGATTT |
|  | MFAP2_BSP_R | TCAATCCTATCAAATACTATCTTATATACA |
|  | MFAP2_MSP_M_F | TTAAGTTGTAATTTTAAGAAATCGT |
|  | MFAP2_MSP_M_R | CAATAATTCAAACAAAAAACGTA |
|  | MFAP2_MSP_U_F | GTGAGATTAAGTTGTAATTTTAAGAAATTG |
|  | MFAP2_MSP_U_R | CTAACAATACAATAATTCAAACAAAAAACA |
| PTPN22 | PTPN22_BSP_F | TTTTTTGGTTTATGTTGTAGAGTAAGAAA |
|  | PTPN22_BSP_R | AAAATAATCTCAATTAAACAAACCACACT |
|  | PTPN22_MSP_M_F | TTTTTTGGTTTATGTTGTAGAGTAAGAAA |
|  | PTPN22_MSP_M_R | CTTCAACATACTCTACTCAAACGAC |
|  | PTPN22_MSP_U_F | TTTTTTGGTTTATGTTGTAGAGTAAGAAA |
|  | PTPN22_MSP_U_R | CTTCAACATACTCTACTCAAACAAC |
| SPNS3 | SPSN3_BSP_F | TGAAGTTGTAGGTTAGATTAAGGGG |
|  | SPSN3_BSP_R | TCACTTCCTATAAAATCCCAAACTC |
|  | SPSN3_MSP_M_F | GGTGTTAGGAGTTTTTGTTTTTC |
|  | SPSN3_MSP_M_R | ACACCTACCCTAACTATTAAACGTC |
|  | SPSN3_MSP_U_F | GGTGTTAGGAGTTTTTGTTTTTTGT |
|  | SPSN3_MSP_U_R | ACACCTACCCTAACTATTAAACATC |
| AIM2 | AIM2_BSP_F | AGAGAATTTGAAAATGAGATTTAAG |
|  | AIM2_BSP_R | AATTAACCATCTTTAATCAAAAAAAA |
|  | AIM2_MSP_M_F | GAGAATTTGAAAATGAGATTTAAGC |
|  | AIM2_MSP_M_R | CCTTCATTATACTTATTCATATAATACGTA |
|  | AIM2_MSP_U_F | GAGAATTTGAAAATGAGATTTAAGTGA |
|  | AIM2_MSP_U_R | CCTTCATTATACTTATTCATATAATACATA |
| SLAMF1 | SLAMF1_BSP_F | GGTTGTTTGATTTATGTATTAGGAATTAGT |
|  | SLAMF1_BSP_R | AAAATAATCCAATAAAACATCTTTTCTTTA |
|  | SLAMF1_MSP_M_F | GGTTTTGGGTAGAAATATGCGT |
|  | SLAMF1_MSP_M_R | TACAATCCAAAAAAACTTACCGAA |
|  | SLAMF1_MSP_U_F | GGTTTTGGGTAGAAATATGTGT |
|  | SLAMF1_MSP_U_R | CTTTACAATCCAAAAAAACTTACCAA |
| SPATA13 | SPATA13_BSP_F | AGTATTTGATGGTTTGTGAGTGTGT |
|  | SPATA13_BSP_R | CCTTCAAAACAAAACTAAAATATAACC |
|  | SPATA13_MSP_M_F | GTTTTGGTCGGGAACGTATATAAC |
|  | SPATA13_MSP_M_R | ACTAAAATATAACCGAAAAACCGAT |
|  | SPATA13_MSP_U_F | TTTGGTTGGGAATGTATATAATGT |
|  | SPATA13_MSP_U_R | ACTAAAATATAACCAAAAAACCAAT |
