# Supplementary material for: Genome-wide DNA methylation profile identified a unique set of differentially methylated immune genes in oral squamous cell carcinoma patients in India
Source: Clin Epigenetics. 2017 Feb 3;9:13. doi: 10.1186/s13148-017-0314-x (PMC5292006; doi:10.1186/s13148-017-0314-x)
Supplement: Additional file 6: Table S6. — Primer sequences used for gene expression study. (DOCX 13 kb) [file 13148_2017_314_MOESM6_ESM.docx]

**Table S3: Primer sequences used for gene expression studies**

| GENE | PRIMER NAME | SEQUENCE |
| --- | --- | --- |
| LXN | E_LXN_F | AAGGTCAAACAAGCCAGCAT |
|  | E_LXN_R | TGTCCCGTTGAAGGGTAAAG |
| PTPN22 | E_PTPN22_F | GCTGTGGAAGGACTGGTGTT |
|  | E_PTPN22_R | TCCCGGATCAAACTGAAAAC |
| CD86 | E_CD86_F | TGGAACCAACACAATGGAGA |
|  | E_CD86_R | TTAAAAACACGCTGGGCTTC |
| CTLA4 | E_CTLA4_F | CTCAGCTGAACCTGGCTACC |
|  | E_CTLA4_R | CTGCTGGCCAGTACCACAG |
| ZNF154 | E_ZNF154_F | ATGTGGCCGTACACTTCTCC |
|  | E_ZNF154_R | TTTTTCTCCAAGGTGCTGCT |
| CTDSP1 | E_CTDSP1_F | ATTACTCAGATCAGCAAGGAGGAGG |
|  | E_CTDSP1_R | CAGAAGAGTGAGTGGAGGATGC |
| ZNF577 | E_ZNF577_F | AAGCCAGATTCGCTCTTCAA |
|  | E_ZNF577_R | CTCCCATATCCACCAAATGC |
| ZSCAN31 | E_ZSCAN31_F | CAAGCAGGAACCAACAGACA |
|  | E_ZSCAN31_R | CTTTGATGCCAACTCCTGGT |
| LDLRAD4 | E_LDLRAD4_F | GTGACCACCTGAACAACAGC |
|  | E_LDLRAD4_R | TTCAGCAGGCAGACGATGAC |
| HLA DPB1 | E_HLA_DPB1_F | GACCTTCCAGATCCTGGTGA |
|  | E_HLA_DPB1_R | TACTCCGGGCAGAATCAGAC |
| RUNX1 | E_RUNX1_F | CACTGCCTTTAACCCTCAGC |
|  | E_RUNX1_R | CAATGGATCCCAGGTATTGG |
| IL6 | IL6_F | GAAAGCAGCAAAGAGGCACT |
|  | IL6_R | TTTCACCAGGCAAGTCTCCT |
| CD28 | E_CD28_F | GCTGCTCTTGGCTCTCAACT |
|  | E_CD28_R | GCTAAGGTTGACCGCATTGT |
| CD80 | E_CD80_F | GCTGGCTGGTCTTTCTCACT |
|  | E_CD80_R | TGCCAGTAGATGCGAGTTTG |
| TLR1 | E_TLR1_F | AGTTGTCAGCGATGTGTTCG |
|  | E_TLR1_R | CCATGCGTGTACCAGACACT |
| TNF | E_TNF_F | CCCGAGTGACAAGCCTGTAG |
|  | E_TNF_R | GAGGTACAGGCCCTCTGATG |
| RPP30 | E_RPP30_F | CAATTTCCAGTGCCCTCAAT |
|  | E_RPP30_R | GCCTAGATTTGCCACGTCAT |
